# CDK4/6 inhibitor ribociclib and doxorubicin combination treatment inhibits breast cancer bone metastasis and enhances T-cell targeted therapy

**DOI:** 10.1016/j.jbo.2026.100765

**Published:** 2026-04-25

**Authors:** Xinming Su, Takayuki Kobayashi, Jingyu Xiang, Yalin Xu, Ayesha N. Shajahan-Haq, Mahta Mardani, Suleyman Noordeen, Kaylee O’Donnell, Kristin A. Kwakwa, Dennis Guan, Gregory C. Fox, Francesca Fontana, Emily Cybulla, Suzanne Bakewell, Alessandro Vindigni, Deborah J. Veis, Samuel Achilefu, Gregory M. Lanza, Katherine N. Weilbaecher

**Affiliations:** a Department of Medicine, Washington University School of Medicine, St. Louis, MO 63110, USA; b Department of Breast Medical Oncology, Cancer Institute Hospital of the Japanese Foundation for Cancer Research, Tokyo, Japan; c Department of Oncology, Lombardi Comprehensive Cancer Center, Georgetown University Medical Center, Washington, DC 20057, USA; d Department of Internal Medicine, The Ohio State University College of Medicine, Columbus, OH 43210, USA; e Siteman Cancer Center, Washington University School of Medicine, St. Louis, MO 63110, USA; f Musculoskeletal Research Center, Histology and Morphometry Core, Washington University School of Medicine, St. Louis, MO 63110, USA; g Department of Pathology and Immunology, Washington University School of Medicine, St. Louis, MO 63110, USA; h Department of Biomedical Engineering, University of Texas Southwestern Medical Center, Dallas, TX 7530, USA; i Department of Cell Biology and Physiology, Washington University School of Medicine, St. Louis, MO 63110, USA

## Abstract

CDK4/6 inhibitors (CDK4/6i) combined with endocrine therapy, specifically aromatase inhibitors or selective estrogen receptor degraders or modulators, have significantly improved outcomes for advanced estrogen receptor-positive (ER^+^) breast cancer; however, therapeutic resistance remains a primary cause of mortality. Overcoming CDK4/6i treatment resistance is an urgent problem. Here, we demonstrate in ER^+^ PyMT-BO1 and triple-negative 4T1 murine models that ribociclib (LEE011) monotherapy reduces primary mammary fat pad (MFP) tumor burden through a mechanism partially dependent on CD8^+^ T cell function. However, LEE011 monotherapy fails to decrease tumor burden in bone colonization models, indicating site-specific resistance within the bone microenvironment. We identified doxorubicin as a synergistic partner that, when combined with LEE011, inhibits tumor cell proliferation and suppresses myeloid-specific arginase 1 (ARG1) expression. While doxorubicin monotherapy reduces tumor burden, its efficacy in bone is often compromised by off-target bone loss and pro-metastatic TGF-β signaling. Notably, the combination of LEE011 and doxorubicin successfully reduces bone metastatic burden, reverses treatment-induced osteoclast activity, and decreases the infiltration of ARG1^+^ immunosuppressive myeloid cells. Furthermore, this combination therapy remodels the bone niche to significantly enhance the efficacy of adoptive T cell immunotherapy. Collectively, these results suggest that the synergy between CDK4/6i and doxorubicin represents a promising therapeutic strategy to overcome the protective signals of the bone microenvironment in metastatic breast cancer.

## Highlights

• Ribociclib monotherapy inhibits primary tumors but fails to reduce bone metastatic burden in breast cancer models.
• Single-agent ribociclib increases peripheral CD8^+^ T-cell abundance and contributes to primary tumor growth inhibition.
• Ribociclib and doxorubicin synergistically inhibit tumor cell proliferation via a DNA repair-independent mechanism.
• The combination therapy reduces bone tumor burden and reverses osteoclast activity and ARG1^+^ myeloid cell infiltration.
• Combined therapy remodels the bone microenvironment to significantly enhance adoptive T-cell therapy efficacy.

## 1 Introduction

Uncontrolled cell cycle progression is a hallmark of malignancy [1]. In healthy cells, growth factor signaling triggers the binding of D-type cyclins (D1, D2, and D3) to cyclin-dependent kinases 4 and 6 (CDK4/6). This complex phosphorylates the retinoblastoma (Rb) protein, releasing E2F transcription factors that drive the transition from G1 to S phase [2]. In breast cancer, overexpression or amplification of this pathway frequently disables these “brakes,” leading to unchecked proliferation.

While CDK4/6 inhibitors (CDK4/6i) combined with endocrine therapy, like aromatase inhibitors, are now the first-line treatment for advanced hormone receptor-positive breast cancer, clinical utility is limited by the eventual development of treatment resistance [3]. Current research to overcome this resistance focuses on identifying predictive biomarkers and exploring combination therapies with targeted agents or chemotherapy [3], [4], [5].

The bone microenvironment presents a unique therapeutic challenge. Compared to primary tumors or visceral metastases, bone metastatic tumors are notably more resistant to both immunotherapy [6], [7], [8], [9] and chemotherapy [4], [7]. Emerging evidence suggests that CDK4/6i treatment efficacy is inconsistent in bone [7], [10], likely due to the complex immune regulation within the bone marrow. Primary breast cancer cells can remotely reprogram bone marrow hematopoiesis, leading to the accumulation of myeloid cells that promote metastatic growth [11], [12]. Although inhibiting the CDK4/6 pathway can modulate the immune system, by reducing regulatory T cells and myeloid derived suppressor cells (MDSCs) [13], and enhancing T cell activation [14], [15], [16], [17]. However, the specific mechanisms of resistance within the bone remain poorly understood. Addressing this efficiency gap is critical for improving outcomes in patients with bone metastatic breast cancer.

In this study, using breast cancer murine models, we observed that bone-colonized breast cancer cells exhibit greater resistance to CDK4/6 inhibition compared to primary tumors. We screened anti-breast cancer therapies that could synergize with ribociclib (LEE011), and identified that doxorubicin could synergize with LEE011 to inhibit tumor cell proliferation via a DNA repair independent mechanism. In PyMT-BO1 and 4T1 tumor cell derived in vivo models, the combination of doxorubicin and LEE011 treatment showed promising results in decreasing visceral and bone tumor burden; and significantly enhanced the tumor-specific T-cell therapy efficacy to decrease bone tumor burden and prolong survival.

## 2 Materials and Methods

### 2.1 Mice

All animal studies were performed according to the guidelines established by the Washington University, Institutional Animal Care and Use Committee (WU IACUC). WT C57BL/6 mice (JAX 000664), BALB/c mice (JAX 000651) and OT-1 mice (JAX 003831) are from the Jackson Laboratory. For in vivo experiments, 8 to 12-week-old female mice were used. Mice used for in vitro experiments were 6 to 12 weeks old female mice. All mice are housed under pathogen-free conditions according to the guidelines of the WU IACUC.

### 2.2 Cell lines and reagents

The BALB/c background 4T1-GFP-Luc (4T1-G-Luc) murine mammary tumor cell line was originally from Dr. David Piwnica-Worms (The University of Texas, Houston, TX) as previously described [18]. The C57BL/6J background PyMT-B6, PyMT-BO1, and PyMT-BO1-GFP-Luc (BO1-G-Luc) murine mammary tumor cell line was previously reported [19]. Both murine breast tumor cell lines had been previously modified to express firefly luciferase (Luc) and green fluorescent protein (GFP). OVA_257–264_ expressing BO1-G-Luc cell line (BO1-G-Luc-OVA) was described previously [20]. All cell lines were maintained in Dulbecco’s Modified Eagle's Medium (DMEM) (Gibco, Waltham, MA, USA) supplemented with 10% fetal bovine serum (Sigma, St. Louis, MO, USA) and 100 ug/mL penicillin streptomycin (Gibco, Waltham, MA, USA). All cell lines tested negative for mycoplasma.

To generate primary bone marrow macrophages (BMM), whole BM was extracted from the femurs and tibias of mice, plated in Petri dishes in DMEM containing 10% FBS and 50 ng/mL M−CSF, and cultured in a 37°C, 5% CO2 incubator. Day 3 cultured BMMs were plated at 5 × 10^5^ cells per well in 6-well cell culture plates and treated with tumor conditioned media or co-cultured with tumor cells at 1:1 ratio for 24 h before analysis [20].

Abemaciclib (LY2835219) was purchased from Cayman Chemical Company (Ann Arbor, MI, USA). Palbociclib (PD0332991), ribociclib (LEE011), 4-Hydroxytamoxifen and ICI182,780 (fulvestrant) were purchased from Selleck Chemicals (Houston, TX, USA). Abemaciclib was dissolved in ethanol. All other drugs were dissolved in dimethyl sulfoxide (DMSO). For in vitro assays, the negative control was 0.02% ethanol or DMSO. Ribociclib (LEE011) used for in vivo experiment was provided by Novartis (Basel, Switzerland). All other reagents were purchased from Sigma Aldrich.

### 2.3 Mice tumor models

4T1-G-Luc cells were implanted into BALB/C mice, and BO1-G-Luc cells were implanted into C57BL/6 mice. In vivo orthotopic breast tumor models were established by injection of 1x10^5^ tumor cells, mixed with BD matrigel (BD biosciences, Franklin Lakes, NJ, USA) or with PBS in a total of 40 µL, into the fourth mammary fat pad (MFP) tissue of 8-week-old female mice. Breast tumor bone metastasis models were established by intracardiac injection with 1x10^5^ tumor cells in 50 µL PBS into 6-week-old mice, as previously described [21]. Bioluminescence imaging was used to quantify tumor growth after injection. For CDK4/6 inhibitor and chemotherapy treatment, mice were given LEE011 (75 mg/kg, Novartis) by oral gavage and doxorubicin (4 mg/kg, Sigma) by intravenous injection at the indicated time points described in figures.

Therapeutic administration for the tumor models commenced on days 3 to 5 post-inoculation once tumors became palpable or detectable. A ribociclib (LEE011) dosage of 75 mg/kg was selected to provide a human equivalent dose (∼427 mg/day), aligning with the clinically established therapeutic range (400–600 mg). Experiments concluded when control tumors reached the institutional ethical limit of 2.0 cm in diameter (MFP models) or upon the onset of mobility-related symptoms (bone models).

For adoptive T cell treatment experiments, OT-1 T cells were expanded in vitro before intravenous injection. Briefly, splenic cells from OT-1 mice were harvested and stimulated with 0.5 μg/mL OVA peptide and 10 ng/mL IL-2 in a T-75 flask, and the media were refreshed every day with IL-2 for 3 days. On day 4, T cells were harvested and CD8^+^ T cells were prepared for injection as previously described [20].

### 2.4 In vivo bioluminescence imaging

For bioluminescence imaging of live animals, as previously described [21], mice were injected intraperitoneally with 150 µg/g D-luciferin (Biosynth, Naperville, IL) in PBS, anesthetized with 2.5% isoflurane, and imaged with a charge-coupled device (CCD) camera-based bioluminescence imaging system (IVIS 100; Caliper, Hopkinton, MA; exposure time 1–60 s, binning 8, field of view 12, f/stop 1, open filter, anterior side). Signal was displayed as photons/sec/cm2/sr. Regions of interest (ROI) were defined manually around the legs using Living Image Igor Pro Software (Version 2.50, Xenogen Corporation; Alameda, CA, USA).

### 2.5 Micro-computed tomography

Tibial metaphysis was scanned by micro-computed tomography (μCT) (μCT-40; Scanco Medical, Brüttisellen, Switzerland). The trabecular region from forty 2D slices (0.8 mm) in the primary and secondary spongiosa of the tibia was selected using contours inside the cortical shell on each 2D image, with the growth plate as a marker to determine a consistent location to start analysis. A 3D cubical voxel model of bone was built, and calculations were made for BV/TV and apparent BMD (calibrated against a hydroxyapatite phantom). A threshold of 300 (out of 1,000) was used to differentiate trabecular bone from nonbone.

### 2.6 Immunohistochemistry

Primary mammary tumors and hind limbs were harvested at the experimental endpoint and fixed in 10% neutral buffered formalin for 48 h at room temperature. Bone specimens were decalcified in EDTA solution for 10 days at room temperature with regular solution changes, followed by routine dehydration and paraffin embedding. Paraffin sections were cut at 5 μm thickness and mounted on glass slides. Sections were deparaffinized, rehydrated through graded ethanol, and stained with hematoxylin and eosin (H&E) using standard protocols.

Histologic slides were imaged on Olympus NanoZoomer 2.0-HT System (Hamamatsu Photonics; Hamamatsu City, Japan). Tumor burden within bone sections was quantified on H&E-stained slides. Digital images were acquired at low magnification, and tumor regions were manually delineated by drawing boundaries around the tumor area. Tumor surface area was measured within the defined region using HALO image analysis software (Indica Labs; Albuquerque, NM, USA). Measurements were performed on representative sections per animal, and tumor area values were averaged per mouse prior to statistical analysis.

Tartrate-resistant acid phosphatase (TRAP) staining was performed on 5 μm paraffin-embedded, decalcified bone sections to assess osteoclast activity. Following deparaffinization and rehydration, sections were incubated in TRAP staining solution according to the manufacturer’s instructions (Sigma-Aldrich; St. Louis, MO, USA). TRAP-positive cells were identified by red cytoplasmic staining. Regions of interest (ROIs) encompassing the tumor–bone interface were selected for analysis. Quantification of TRAP positive cells within selected ROIs was performed using HALO image analysis software (Indica Labs; Albuquerque, NM, USA). Osteoclasts were defined as TRAP positive multinucleated cells located along the bone surface. The number of TRAP positive cells per ROI was calculated and averaged per animal for downstream statistical analysis.

Ki67 and ARG1 immunohistochemical staining was performed on paraffin-embedded sections (5 μm) from both primary mammary tumors and bone metastatic lesions. Following deparaffinization and rehydration, antigen retrieval was carried out in Tris-EDTA buffer (pH 8.0) using a steamer. Endogenous peroxidase activity was quenched prior to incubation with primary antibody. Sections were incubated with rabbit monoclonal anti-Ki67 antibody (Cell Signaling Technology, clone D3B5; Danvers, MA, USA) or anti-ARG1 antibody (Cell Signaling Technology, clone D4E3M) at a dilution of 1:200. Detection was performed using an HRP-based system with diaminobenzidine (DAB) as chromogen. Slides were counterstained with hematoxylin, dehydrated, and coverslipped. Whole-slide images were acquired, and five representative regions of interest (ROIs) per sample were selected for analysis. Quantification of Ki67-positive nuclei or ARG1-positive cells were performed using HALO image analysis software (Indica Labs). The percentage of Ki67-positive or ARG1-positive cells was calculated within each ROI and averaged per sample. All analyses were performed in a blinded manner.

### 2.7 Western blot

Cultured tumor cells were washed once with cold 1x PBS and lysed with radio-immunoprecipitation assay (RIPA) buffer supplemented with PhosSTOP phosphatase and CompleteMini protease inhibitors (Roche, Switzerland) for protein extraction. Proteins were separated by polyacrylamide gel electrophoresis using 4 − 12% gradient gels followed by protein transfer onto nitrocellulose membranes with iBLOT2 (Thermo Fisher Scientific, Waltham, MA, USA). Membranes were blocked in 5% nonfat dry milk made in Tris-buffered saline with Tween-20 (TBST) and rocked at 4°C with the respective primary antibodies overnight. Secondary antibodies conjugated with horseradish peroxidase and SuperSignal West Dura Extended Duration Substrate (Thermo Fisher Scientific) were used to detect proteins of interest using a GE Amersham 600 RGB gel imager. Antibodies for estrogen receptor alpha (ER) (#ab108398) was from Abcam, for progesterone receptor (PR) was from ThermoFisher (MA1-411) and for HER2 (#2165) was purchased from Cell Signaling. Actin (#sc-47778) antibody was purchased from Santa Cruz Biotech (Dallas, TX, USA). Vinculin (#4650) and Phospho-histone H2AX (#2577) antibodies, and secondary antibodies for mouse (#7076) and rabbit (#7074) were purchased from Cell Signaling Technology (Danvers, MA, USA).

### 2.8 In vitro synergy killing assay

5000 BO1-GFP-Luc cells were plated in 96-well-plates overnight, followed by treatment with various doses of LEE011, in the presence or absence of chemotherapy drugs (Doxorubicin, Eribulin, Cisplatin, Bortezomib, Etoposide; all from Sigma, St. Louis, MO, USA) for 72 hr. Cell viability was assessed via luciferase assay on a GloMax Luminometer (Promega; Madison, WI, USA). Synergy scores were calculated using the ZIP or Bliss model from the SynergyFinder package [22].

### 2.9 Flow cytometry

Tumor tissues were prepared in single-cell suspensions for FACS analysis. Briefly, MFP tumor tissue was manually minced using a scalpel or scissors, followed by enzymatic digestion with 1 mg/mL collagenase A (Roche, Indianapolis, IN, USA) and DNase I (Sigma-Aldrich, St. Louis, MO, USA) for 30 min at 37°C with constant stirring. Cells were filtered through 100 μm nylon strainers (Thermo Fisher Scientific), and then washed twice in PBS with 2% FBS. To isolate splenocytes, spleens were harvested and mechanically dissociated through a 100 µm cell strainer using a syringe plunger to create a single-cell suspension. Bone marrow cells were extracted by flushing the cavities of the femur and tibia with cold PBS using a 25G needle. Both cell populations were subjected to ACK lysis buffer to remove red blood cells, washed, and filtered before being resuspended in flow cytometry buffer for staining. After counting, 1 × 10^6^ total cells were placed in 100 μL buffer (PBS plus 2% FBS, 2 mM EDTA) and incubated for 20 min with fluorophore-conjugated anti-mouse antibodies using the manufacturers’ recommended concentrations. All antibodies used are previously described [20] and listed in supplementary Table S1. Data acquisition was performed on an LSR-II or X20 system (BD Biosciences; Franklin Lakes, NJ, USA), and FlowJo software (version 10, FlowJo, LLC; Ashland, OR, USA) was used for analysis.

### 2.10 Cell proliferation by crystal violet assay

For PyMT-B6 and PyMT-BO1 cell proliferation studies, cells were seeded with 1000 cells per well in 96-well plastic tissue culture plates per cell line. After 24 h, cells were dosed with 0.02% vehicle (ethanol or DMSO) or various concentrations of the indicated drugs for 6 days. For crystal violet staining, plates were rinsed once with PBS followed by staining with 100  μL crystal violet at room temperature for 1  h. Excess stain was removed and plates were rinsed 5 − 10x with H_2_O. The plates were left at room temperature to dry overnight, then were rehydrated with 100  μL 0.1  M sodium citrate buffer in 50% ethanol. Plates were measured using a Vmax kinetic microplate reader (Molecular Devices; San Jose, CA, USA) with an absorbance of 560  nm. Results were normalized to vehicle. All experiments were repeated at least three times with n = 5–10 technical replicates for each condition.

### 2.11 MTT viability assay

For BO1-G-Luc and 4T1-G-Luc cell proliferation studies, a total of 5,000 cells per well were plated in 96-well plates with indicated concentrations of drugs. After 72 h, MTT (3-(4,5-dimethylthiazol-2-yl)-2,5-diphenyltetrazolium bromide) (Sigma, St. Louis, MO USA) was added for 4 h at a final concentration of 0.2 mg/ml. After remove the media, 150 uL DMSO was added, and plates were measured using a Vmax kinetic microplate reader (Molecular Devices; San Jose, CA, USA) to measure absorbance at 570 and 630 nm.

### 2.12 Cell cycle analysis

BO1-G-Luc tumor cells were seeded at a density of 2x10^5^ cells per well in 6-well plates and treated with 1 uM LEE011, 15 nM doxorubicin, or their combination for 24 h. Following treatment, cells were harvested, washed in ice-cold PBS, and fixed in 70% ethanol for 2 h. For DNA quantification, fixed cells were washed and resuspended in PBS containing 50ug/mL Propidium Iodide (PI) and 100ug/mL RNase A, then incubated in the dark for 30 min at room temperature. Data acquisition was performed on an LSR-II or X20 system (BD Biosciences; San Jose, CA, USA), and FlowJo software (version 10, FlowJo, LLC; Ashland, OR, USA) was used for analysis.

### 2.13 Apoptosis assay

Cell apoptosis was evaluated using Annexin V-PE and 7-AAD staining according to the manufacturer’s instructions (BioLegend; San Diego, CA, USA). Briefly, BO1-G-Luc cells were seeded at 2x10^5^ cells/well and treated with 1 uM LEE011, 15 nM doxorubicin, or the combination for 24 h. Cells were harvested and washed in cold PBS. The cell pellets were resuspended in 1X Binding Buffer and incubated with Annexin V-PE and 7-AAD for 15 min in the dark at room temperature. Samples were analyzed immediately using the BD X20 flow cytometer. Apoptotic populations were classified as Annexin V + with data processed using FlowJo software (version 10; FlowJo, LLC, Ashland, OR, USA).

### 2.14 Statistics

GraphPad Prism Version 10 (GraphPad Software; Boston, MA, USA) was used for statistical analyses. Differences between two groups were evaluated by an unpaired two-tailed Student’s *t* test, while multi-group comparisons were analyzed via one or two-way repeated measures ANOVA followed by Tukey’s post-hoc test for multiple comparisons. Data are presented as mean ± SEM, with significance defined as *p* < 0.05.

### 2.15 Study approval

Animal work was performed according to the policies of the WU IACUC at Washington University School of Medicine in St. Louis. Mice were analyzed under approved protocols and were provided appropriate care while undergoing research that complied with the standards in the Guide for the Use and Care of Laboratory Animals (National Academies Press, 2011) and the Animal Welfare Act.

## 3 Results

### 3.1 LEE011 monotherapy reduces primary breast tumor growth but fails to decrease bone metastatic burden

**Fig. 1:**
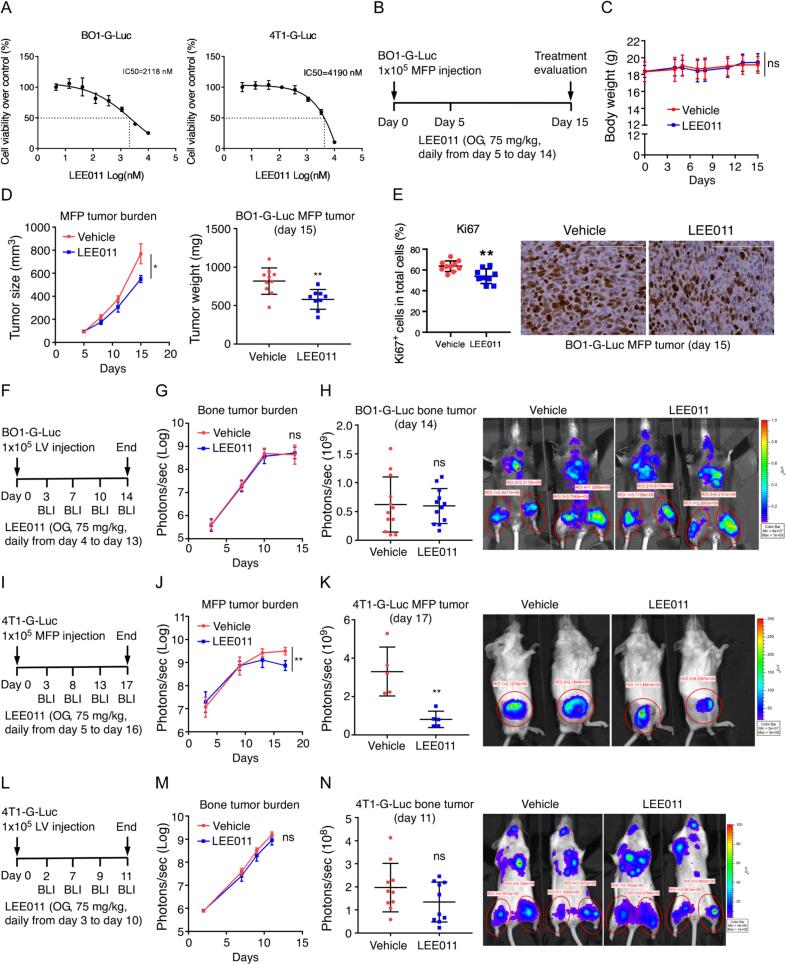
**LEE011 monotherapy reduces primary breast tumor growth but fails to decrease bone metastatic burden. (A)** In vitro cell viability of ER^+^ Luminal B BO1-G-Luc and TNBC 4T1-G-Luc cell lines assessed by MTT assay. **(B)** Experimental schema for the mammary fat pad (MFP) model: 8-week-old female C57BL/6J mice were injected with BO1-G-Luc cells (1 × 10^5^) and treated with ribociclib (LEE011) daily via oral gavage (Days 5 to 14). **(C)** Mice body weight measurements. **(D)** Primary tumor kinetics measured by calipers and final tumor weight at Day 14. **(E)** Quantification of Ki67^+^ proliferating cells in BO1-G-Luc MFP tumor sections. **(F)** Experimental schema for the bone colonization model: 6-week-old female C57BL/6J mice were intracardially injected with BO1-G-Luc cells and treated with LEE011 (Days 4 to 13). **(G, H)** Bioluminescence imaging (BLI) quantification and representative BLI images of bone tumor burden at Day 14. **(I)** MFP model in BALB/c mice: 8-week-old females were injected with 4T1-G-Luc cells and treated with LEE011 (Days 5 to 16). **(J, K)** MFP tumor growth quantified by BLI and representative images at Day 17. **(L)** Bone colonization model in BALB/c mice: 6-week-old females were intracardially injected with 4T1-G-Luc cells and treated with LEE011 (Days 3 to 10). **(M, N)** Bone tumor burden quantified by BLI and representative images at Day 11. Data are presented as mean ± SEM. Panels (C, D, G, J, M) were analyzed by one-way ANOVA with Tukey’s post-hoc test; all other panels were analyzed by two-tailed, unpaired Student’s *t*-test with Welch’s correction. *P < 0.05, **P < 0.01.

We employed two immunocompetent, RB1 intact, endocrine therapy-resistant murine breast cancer models, ER + luminal B PyMT-BO1 and triple-negative 4T1 tumor models, to evaluate the efficacy and immunomodulatory effects of the CDK4/6 inhibitor (CDK4/6i) ribociclib (LEE011). PyMT-BO1 cells express estrogen (ER), progesterone (PR), and Her2 receptors but exhibit in vitro resistance to tamoxifen and fulvestrant (Fig. S1). For in vivo tracking, we utilized GFP- and luciferase-labeled variants (BO1-G-Luc and 4T1-G-Luc). LEE011 treatment reduced cell viability in both lines, with IC50 values of 2118 nM (BO1-G-Luc) and 4190 nM (4 T1-G-Luc), representing a relatively resistant phenotype compared to highly sensitive lines with nanomolar IC50s **(**Fig. 1**A)**.

To assess monotherapy activity, we established mammary fat pad (MFP) tumors using BO1-G-Luc cells in C57BL/6J mice. Daily oral gavage of 75 mg/kg LEE011 commenced on day 5 (average tumor size 100 mm^3^). Despite their relative in vitro resistance, single-agent LEE011 significantly suppressed in vivo MFP tumor growth **(**Fig. 1**B-D)**. Immunohistochemical analysis confirmed this effect, showing a significant, albeit modest, reduction in Ki67^+^ proliferating cells **(**Fig. 1**E)**, consistent with cell cycle blockade.

We next evaluated LEE011 efficacy in the bone, the most frequent site of breast cancer metastasis and a niche known for therapeutic resistance. BO1-G-Luc cells were injected intracardially to establish bone colonization, followed by 9 days of LEE011 treatment. Notably, bone-colonized tumors showed no response to LEE011 **(**Fig. 1**F-H)**. Given that enhanced osteoclast function can promote tumor growth and counteract CDK4/6 inhibition [23], we investigated whether LEE011 altered bone homeostasis. In vitro, LEE011 inhibited osteoclastogenesis when administered at day 0 but had no effect when added at day 3 during pre-osteoclast fusion **(**Fig. S2**)**. In vivo, 10 days of LEE011 treatment did not significantly alter trabecular bone volume (BV/TV) or bone mineral density (BMD) in non-tumor-bearing mice **(**Fig. S3**)**. These findings suggest that the resistance of bone colonized cells is not primarily driven by direct drug-induced changes to osteoclast function.

These results were corroborated using the 4T1-G-Luc model. While 4T1 primary tumors in the MFP remained sensitive to LEE011 **(**Fig. 1**I-K)**, bone metastatic lesions were entirely resistant **(**Fig. 1**L-N)**. Collectively, these data indicate that the bone microenvironment (BME) provides unique protective signals that confer resistance to CDK4/6i therapy.

### 3.2 LEE011 monotherapy increases CD8^+^ T cell populations in peripheral organs and contributes to the inhibition of primary tumor growth

**Fig. 2:**
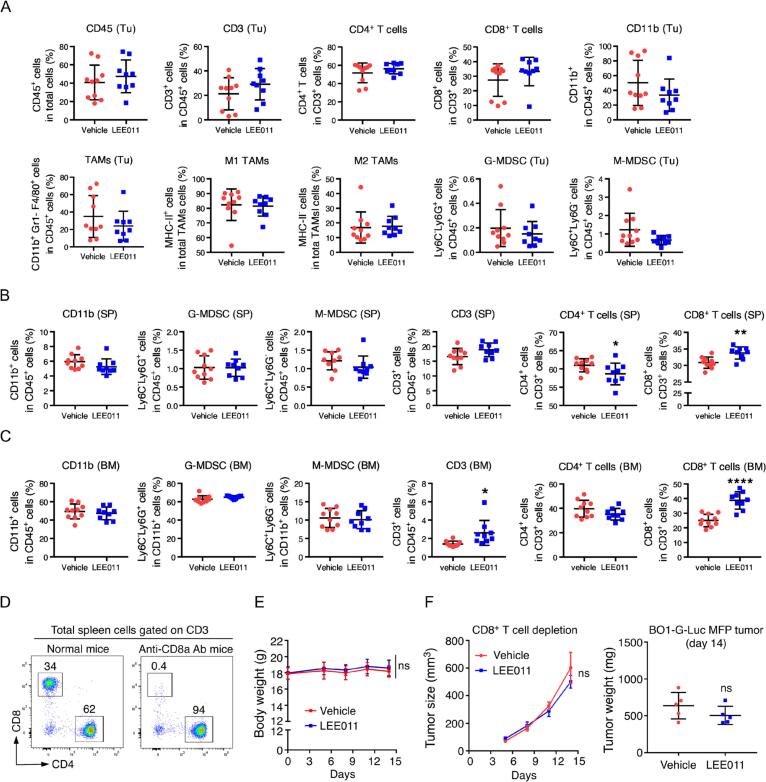
**LEE011 monotherapy increases CD8^+^ T cell numbers in peripheral organs and contributes to the inhibition of primary tumor growth. (A–C)** Immune profiling of the tumor microenvironment and systemic compartments: BO1-G-Luc breast cancer cells (1 × 10^5^) were injected into the mammary fat pad (MFP) of 8-week-old female C57BL/6J mice. Ribociclib (LEE011) was administered by oral gavage from Day 5 to Day 14. On Day 15, **(A)** primary tumor tissue, **(B)** spleen, and **(C)** bone marrow were harvested for flow cytometric analysis (FACS). **(D–F)** CD8^+^ T-cell depletion study: Mice were treated with an anti-CD8α antibody (200 μg/mouse, i.p.) one day prior to tumor injection and on Day 6 post-injection; depletion was confirmed by FACS. **(E)** Mice body weight measurements. **(F)** Primary tumor burden, evaluated by tissue volume (growth curve) and final weight. Tumor size data are presented as mean ± SEM; ns = no significant differences by one-way ANOVA with Tukey’s post-hoc test. All other data are presented as mean ± SEM. *P < 0.05, **P < 0.01, ***P < 0.001, ****P < 0.0001, by two-tailed, unpaired Student’s *t*-test with Welch’s correction.

To identify factors contributing to site-specific CDK4/6i resistance, we investigated systemic and local immune landscapes via flow cytometry. In BO1-G-Luc MFP tumor-bearing mice, we analyzed tumor-infiltrating leukocytes (CD45^+^), T cell subsets (CD4^+^, CD8^+^), and myeloid populations; however, no significant differences were observed between vehicle and LEE011-treated groups within the primary tumor microenvironment (Fig. 2**A**).

Consistent with clinical observations of treatment-induced myelosuppression, we noted a decrease in circulating monocytes. In non-tumor-bearing mice, high-dose LEE011 (150 mg/kg) significantly reduced blood monocyte counts, while the therapeutic dose (75 mg/kg) did not **(**Fig. S4**A)**. Furthermore, the percentage of CD11b^+^ myeloid cells in the bone marrow decreased significantly from 60% in controls to 55% at the low dose and 50% at the high dose. Interestingly, in tumor-bearing mice, myeloid populations in the spleen and bone marrow remained unchanged after treatment. However, we observed a notable shift in the T cell compartment: CD8^+^ T cell percentages (of total CD3^+^ cells) increased significantly in both the spleen (from 30% to 35%) and the bone marrow (from 25% to 40%) **(**Fig. 2**B, 2C)**. A similar expansion of bone marrow CD8^+^ T cells was observed in non-tumor-bearing mice, though only at the high dose **(**Fig. S4**C)**. These results suggest that LEE011 expands bone marrow CD8^+^ T cells independently of tumor presence; however, a tumor-conditioned environment appears to sensitize the bone marrow, enabling robust CD8^+^ T cell increases even at lower therapeutic doses.

To determine the functional requirement of these cells in the therapeutic response, we depleted CD8^+^ T cells using a neutralizing antibody prior to MFP implantation. In the absence of CD8^+^ T cells, LEE011 treatment failed to reduce primary tumor burden **(**Fig. 2**D-F)**. Collectively, these data indicate that the growth inhibitory effect of single-agent LEE011 on primary tumors is CD8^+^ T-cell dependent, yet this immune activation is insufficient to overcome resistance in the bone microenvironment.

### 3.3 LEE011 and doxorubicin synergize and inhibit tumor cell growth via a DNA repair-independent mechanism

**Fig. 3:**
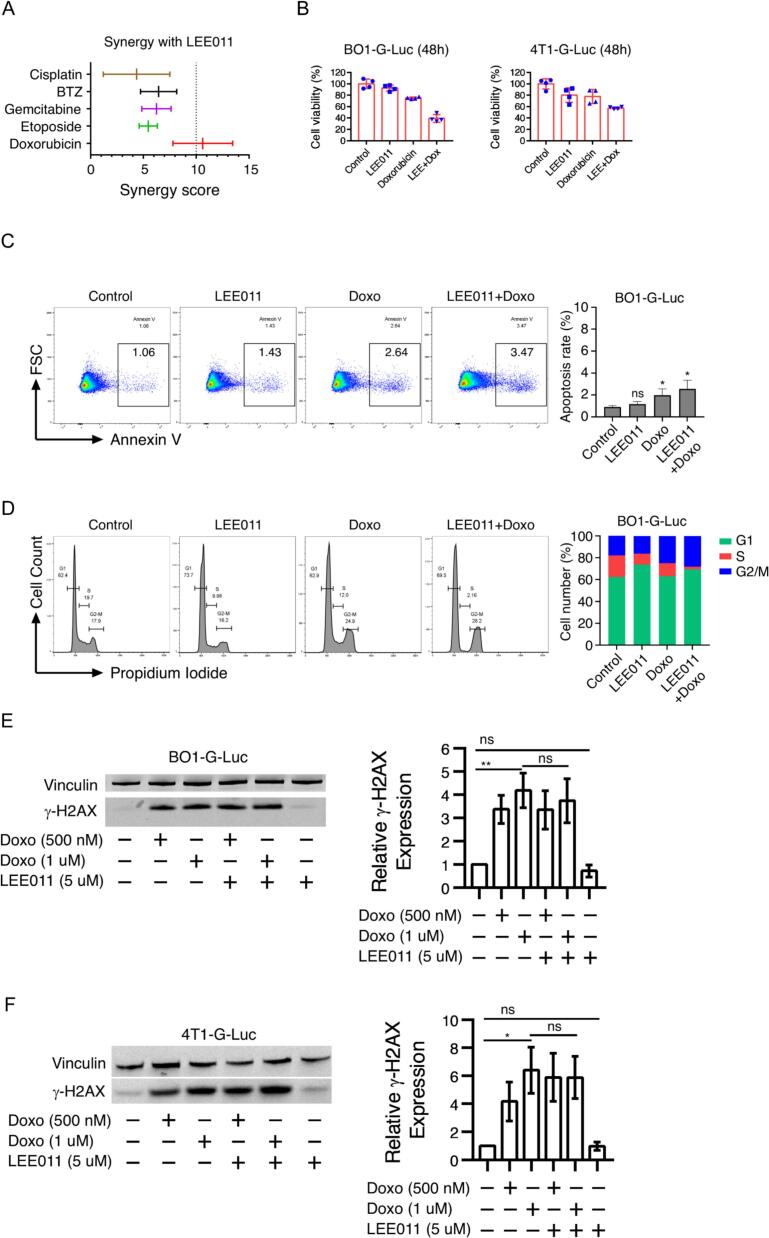
**LEE011 and doxorubicin synergize to inhibit tumor cell growth via a DNA repair-independent mechanism in vitro. (A)** Synergy scores for the combination of ribociclib (LEE011) with various chemotherapeutic agents (doxorubicin, eribulin, cisplatin, bortezomib, and etoposide), determined by cell viability assays. **(B)** BO1-G-Luc and 4T1-G-Luc breast cancer cells were treated with 1 μM LEE011, 15 nM doxorubicin, or the combination for 48 h; cell viability was evaluated using an MTT assay. **(C)** Apoptosis was quantified by Annexin V staining. **(D)** Cell cycle distribution was analyzed by propidium iodide (PI) staining. Data are representative of three independent experiments. **(E, F)** Western blot analysis of DNA damage markers: BO1-G-Luc and 4T1-G-Luc cells were pre-treated with or without LEE011 for 18 h, followed by 6 h of doxorubicin exposure. Data are presented as mean ± SEM. *P < 0.05, **P < 0.01, by two-tailed, unpaired Student’s *t*-test with Welch’s correction.

Since CDK4/6 inhibition typically induces G1 cell cycle arrest, we investigated whether combining ribociclib (LEE011) with various chemotherapeutic agents could further suppress tumor cell proliferation. We screened BO1-G-Luc cells with combinations of LEE011 and several clinical agents, including cisplatin, bortezomib, gemcitabine, etoposide, and doxorubicin. Among the tested compounds, the combination of LEE011 and doxorubicin exhibited the highest synergy scores **(**Fig. 3**A)**. Cell viability assays confirmed this synergistic effect in both BO1-G-Luc and 4T1-G-Luc lines, with the combination therapy reducing tumor cell viability by more than 50% compared to monotherapy **(**Fig. 3**B)**.

To understand the nature of this growth inhibition, we performed apoptosis and cell cycle analyses. While both LEE011 and doxorubicin increased apoptosis, the total percentage of apoptotic cells remained relatively low, suggesting that the primary effect was not immediate cell death **(**Fig. 3**C)**. In cell cycle assays, LEE011 monotherapy induced G1 arrest, while doxorubicin monotherapy primarily arrested cells in G2/M phase. Notably, the combination treatment resulted in a dramatic reduction of cells in S-phase, with a concomitant increase in both G1 and G2/M populations **(**Fig. 3**D)**.

Given that doxorubicin is a potent DNA-damaging agent, we investigated whether LEE011 sensitized cells by impairing DNA repair. Interestingly, western blot analysis of γH2AX expression revealed that LEE011 did not enhance doxorubicin-induced DNA damage in either BO1-G-Luc or 4T1-G-Luc cells **(**Fig. 3**E, 3F)**. Collectively, these data indicate that LEE011 and doxorubicin synergistically inhibit tumor cell proliferation through a mechanism that is independent of enhanced DNA damage, likely driven by simultaneous arrest at multiple cell cycle checkpoints.

### 3.4 Combined LEE011 and doxorubicin treatment reduces bone tumor burden

**Fig. 4:**
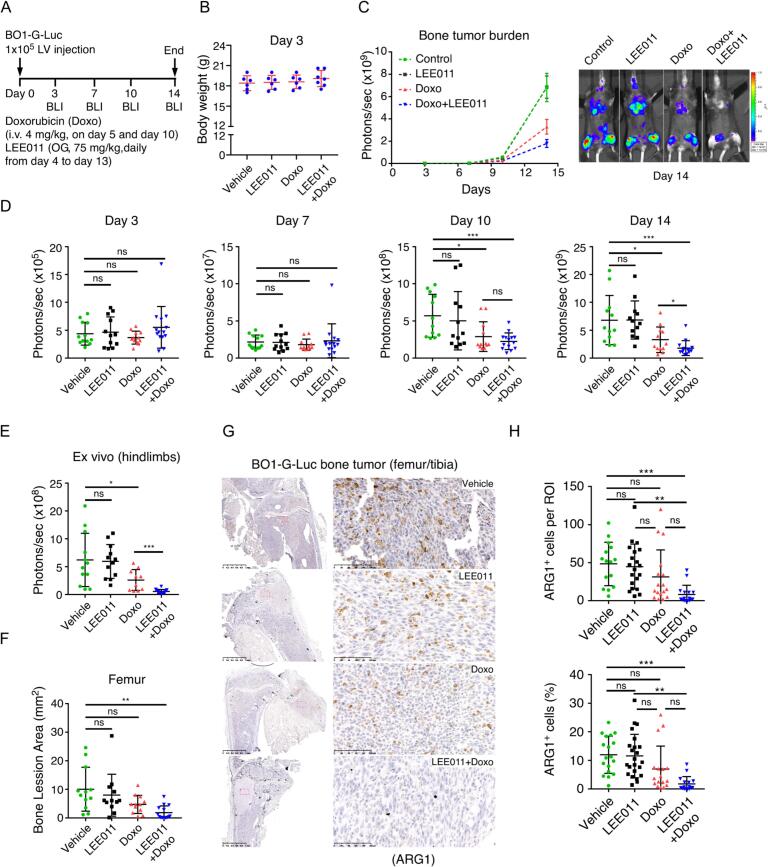
**Combined LEE011 and doxorubicin treatment reduces bone tumor burden and modulates the immune microenvironment. (A)** Experimental schema: PyMT-BO1-GFP-Luc breast cancer cells (1 × 10^5^) were injected intracardially into 6-week-old female C57BL/6J mice. **(B)** Mice body weight measurements. **(C, D)** Mice were treated with ribociclib (LEE011) via oral gavage (Days 4 to 13) and doxorubicin (4 mg/kg) via intravenous (i.v.) injection (Days 5 and 10). Bioluminescence imaging (BLI) was used to quantify systemic and bone tumor burden on Days 3, 7, 10, and 14. **(E)** On Day 14, mice were euthanized for ex vivo BLI to assess metastatic burden in specific organs. **(F)** X-ray analysis and quantification of bone lesion area within the distal femurs. **(G)** Representative arginase 1 (ARG1) immunohistochemical (IHC) staining of bone sections; scale bar = 100 μm. **(H)** Quantification of ARG1 expression within the bone tumor microenvironment. Data are presented as mean ± SD. *P < 0.05, **P < 0.01, ***P < 0.001, by two-tailed, unpaired Student’s *t*-test with Welch’s correction.

To evaluate whether the synergy between ribociclib (LEE011) and doxorubicin translates to the bone microenvironment, we established bone-colonized tumors via intracardiac injection of BO1-G-Luc cells. While no significant differences in tumor burden were observed during the first three days of treatment, by day 10, both doxorubicin monotherapy and the combination therapy significantly reduced bone tumor burden compared to vehicle or LEE011 alone. By day 14, the combination treatment group exhibited significantly lower bone tumor burden than the doxorubicin monotherapy group **(**Fig. 4**A-D)**. Notably, mice receiving the combination therapy experienced less body weight loss than those treated with doxorubicin alone **(**Fig. S5**A)**. Clinical chemistry and hematological analysis revealed that the combination group had decreased white blood cell and neutrophil counts but an increased lymphocyte percentage **(**Fig. S5**B)**. Furthermore, the combination group showed significantly lower blood urea nitrogen (BUN) levels compared to controls, while AST and ALT liver function tests remained within normal ranges **(**Fig. S5**C)**.

The superior efficacy of the combination was further validated by ex vivo BLI and X-ray analysis, which confirmed that the combination treatment group had the lowest bone tumor burden and fewest skeletal lesions **(**Fig. 4**E, 4F)**. Histological analysis revealed a striking shift in bone homeostasis: while LEE011 monotherapy significantly increased TRAP^+^ osteoclast numbers (from 5% to 15%), the addition of doxorubicin reversed this effect **(**Fig. S6**A, B)**. Histomorphometric quantification of the tumor area confirmed that the combination group had the smallest median tumor burden (0.5 mm^2^) compared to doxorubicin (1.5 mm^2^), LEE011 (2.5 mm^2^), and vehicle (3.0 mm^2^) groups **(**Fig. S6**C, D)**. Collectively, these data suggest that the combination therapy not only reduces tumor burden but also maintains a better overall health profile.

We previously demonstrated that ARG1 is predominantly expressed in myeloid cells and inhibits T-cell function within the bone microenvironment [20]. Immunohistochemical analysis of bone tumor sections showed that ARG1^+^ cells decreased significantly from 12% in controls to only 2% in the combination treatment group **(**Fig. 4**G, H)**. To investigate this further, we induced bone marrow macrophages (BMMs) into an ARG1^+^ immunosuppressive state using GM-CSF and lactic acid. While LEE011 monotherapy did not affect ARG1 expression in vitro, the combination treatment significantly reduced the percentage of ARG1^+^ cells from over 60% to approximately 40% **(**Fig. S7**)**. These findings indicate that the synergy between LEE011 and doxorubicin reduces bone tumor burden not only through intrinsic tumoral arrest but also by reducing immunosuppressive myeloid cells within the tumor microenvironment (TME).

### 3.5 Combined LEE011 and doxorubicin treatment enhances the efficacy of adoptive T cell therapy

**Fig. 5:**
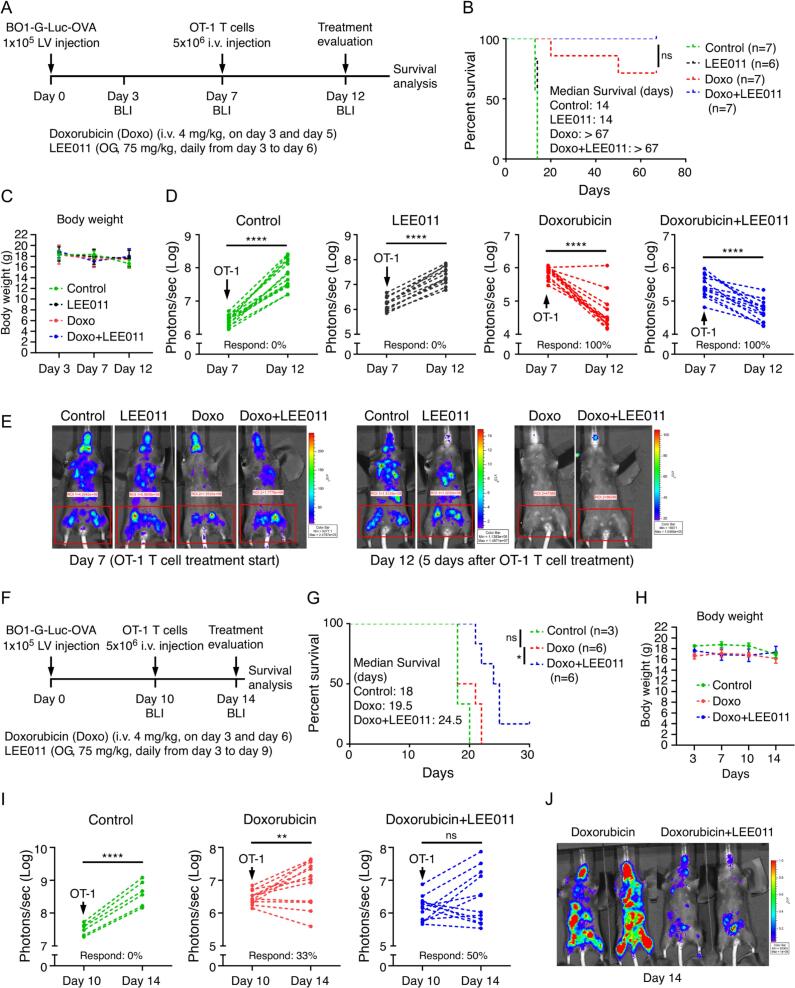
**Combined treatment with LEE011 and doxorubicin enhances the efficacy of adoptive T-cell therapy. (A)** Experimental schema: PyMT-BO1-OVA breast cancer cells (1 × 10^5^) were injected intracardially into 6-week-old female C57BL/6J mice. Ribociclib (LEE011) was administered by oral gavage (Days 3 to 6), and doxorubicin (4 mg/kg) was administered intravenously (Days 3 and 5). OT-I T cells (5 × 10⁶) were delivered intravenously on Day 7. **(B)** Survival analysis following OT-I T cell therapy. **(C)** Body weight measurements. **(D)** T cell treatment response, evaluated by quantifying the change in tumor burden within the same limb before and after T cell administration. The response rate is defined as the percentage of mice exhibiting stabilized or decreased tumor growth (number of responders/total mice × 100%). **(E)** Representative bioluminescence imaging (BLI) of tumor burden. **(F)** Experimental schema for delayed T cell administration: PyMT-BO1-OVA cells were injected as in (A), with LEE011 and doxorubicin administered as indicated, followed by OT-I T cell injection on Day 10. **(G)** Survival analysis following delayed T cell therapy. **(H)** Body weight measurements. **(I)** T cell treatment response calculated as in (D). **(J)** Representative BLI images. Kaplan–Meier curves were used to demonstrate survival profiles, and the log-rank test was used to determine differences between groups. All other data are presented as individual values. *P < 0.05, **P < 0.01, ***P < 0.001, ****P < 0.0001, by two-tailed, unpaired Student’s *t*-test with Welch’s correction.

Given that the combination of ribociclib (LEE011) and doxorubicin reduces ARG1^+^ immunosuppressive myeloid cells, and that LEE011 efficacy is CD8^+^ T cell dependent **(**Fig. 2, Fig. 3, Fig. 4**)**, we hypothesized that this regimen would sensitize bone metastases to T-cell-based immunotherapy. We established bone-colonized tumors using BO1-G-Luc-OVA cells and administered the combination therapy followed by a single dose of activated OT-I T cells on Day 7 **(**Fig. 5**A)**. Therapeutic response was defined as either stabilized or decreased tumor growth post-T-cell transfer. While the vehicle and LEE011 monotherapy groups failed to respond to T cell therapy, both the doxorubicin and combination treatment groups exhibited a 100% response rate. Notably, the combination group achieved 100% overall survival with a median survival exceeding 67 days, significantly outperforming the control and LEE011 groups, which both reached ethical endpoints by Day 14 **(**Fig. 5**B–E)**.

To further differentiate the efficacy between the doxorubicin and combination groups in a more advanced disease setting, we repeated the experiment but delayed OT-I T cell administration until Day 10. In this delayed model, the doxorubicin group showed a 33% response rate with a median survival of 19.5 days. In contrast, the combination treatment group maintained a 50% response rate and a significantly extended median survival of 24.5 days **(**Fig. 5**F–J)**. Kaplan-Meier analysis confirmed that the combination therapy significantly prolonged survival compared to doxorubicin monotherapy. Collectively, these data demonstrate that the synergy between LEE011 and doxorubicin remodels the bone microenvironment to significantly enhance the efficacy of adoptive T cell immunotherapy.

## 4 Discussion

In this study, we demonstrate that bone-colonized breast cancer cells exhibit significantly higher resistance to CDK4/6 inhibition compared to the primary tumor. We further show that combining the CDK4/6 inhibitor ribociclib (LEE011) with doxorubicin exerts a synergistic anti-tumor effect that overcomes this site-specific resistance in murine models.

Primary bone malignancies, such as osteosarcoma, are frequently resistant to CDK4/6 inhibition due to prevalent RB1 pathway mutations [24], [25]. Additionally, tumors can bypass CDK4/6 blockade by upregulating alternative cell cycle regulators, such as CDK2, cyclin E, or cyclin A [26]. Our findings suggest that the resistance observed in breast cancer bone metastases involves not only these intrinsic molecular adaptations but also protective interactions within the bone microenvironment (BME) [27]. The BME is a highly metabolic niche where bone remodeling and host cell signaling intricately interact. Osteoclast activity triggers the release of TGF-β from the bone matrix, which activates the AKT/mTOR pathway, promotes cell-cycle progression, and has been directly linked to CDK4/6 inhibitor resistance [28], [29]. Furthermore, TGF-β signaling fosters an immunosuppressive environment by increasing regulatory T cells (Tregs), cancer-associated fibroblasts (CAFs), and tumor-associated macrophages (TAMs), while upregulating the PD-1/PD-L1 axis [30], [31], [32].

While CDK4/6 inhibitors have demonstrated clinical benefit across various metastatic sites [33], their potential to enhance anti-tumor immunity is an area of intense investigation. Preclinical models have shown that these inhibitors can remodel the tumor microenvironment (TME) by reducing immunosuppressive myeloid-derived suppressor cells (MDSCs) and increasing CD8^+^ T cell infiltration [16], [17]. In our models, we found that the efficacy of LEE011 monotherapy against primary tumors was partially CD8^+^ T cell dependent. Interestingly, while total CD8^+^ T cell numbers did not increase within the primary tumor tissue itself, they significantly expanded in the spleen and bone marrow (Fig. 2). This suggests that LEE011 may enhance the functional status of T cells or their systemic recruitment, even if local densities remain stable. It is possible that the lack of response in the bone following LEE011 monotherapy may be influenced by the initial tumor load at the start of treatment. In our bone colonization model, the rapid establishment of the bone niche may create a threshold of burden that exceeds the inhibitory capacity of a single-agent cell cycle blocker. However, given that similar tumor volumes in the primary mammary fat pad remained sensitive to LEE011, these data suggest that the intrinsic immunosuppressive signaling and protective growth factors unique to the bone microenvironment, rather than tumor mass alone, are the predominant drivers of therapeutic resistance.

The immunomodulatory role of doxorubicin is equally complex. While it can upregulate cytotoxic T-cell pathways via JAK-STAT and TNF-α signaling [34], it also induces PD-L1 expression in tumor and stromal cells, which can paradoxically trigger immune cell apoptosis and limit CD8^+^ T cell proliferation [35], [36]. Research into the combination of doxorubicin and CDK4/6 inhibitors has yielded mixed results [3], [37], [38], [39]. While some studies suggest that inhibitors like palbociclib can increase T-cell and natural killer (NK) cell infiltration [35], others have reported systemic lymphopenia or reduced NK cell activity [40], [41]. Our data help clarify these discrepancies by focusing on the ribociclib (LEE011) specifically. Unlike earlier clinical reports that showed minimal alterations in circulating immune cells [13], [42], our study demonstrates that the LEE011/doxorubicin combination effectively remodels the bone TME by reducing ARG1^+^ immunosuppressive myeloid cells and enhancing the efficacy of adoptive T cell therapy.

While our pilot studies in non-tumor-bearing mice utilized a 150 mg/kg dose of LEE011 to observe the maximal physiological impact on systemic immune populations and bone homeostasis, we intentionally selected the 75 mg/kg dose for our therapeutic synergy models. This lower dose (approximately 6.1 mg/kg human equivalent) more closely aligns with the standard 400–600 mg daily clinical range used in the MONALEESA trials [43], [44], ensuring that our findings remain translationally relevant. Despite the more pronounced monocytopenia observed at the higher dose (Fig. S4), our data (Fig. 4 and Fig. 5) confirm that 75 mg/kg is sufficient to remodel the bone TME and achieve robust synergy with doxorubicin while maintaining a manageable safety profile. Future studies are warranted to evaluate whether even lower, metronomic dosing of both agents could further optimize immune function and anti-tumor activity.

Collectively, our data suggest that the synergy between ribociclib (LEE011) and doxorubicin inhibits metastatic bone tumor burden through both tumor-intrinsic cell cycle arrest and extrinsic immune modulation. By neutralizing the immunosuppressive myeloid compartment and potentiating CD8^+^ T cell function, this combination therapy represents a promising strategy for overcoming the protective signals of the bone microenvironment. Future research should focus on optimizing these regimens to maximize anti-cancer activity while preserving systemic immune health.

## 5 Conclusion

Drug resistance remains a significant barrier in treating breast cancer, particularly within the bone microenvironment where resistance to chemotherapy and immunotherapy is heightened. While CDK4/6 inhibitor monotherapy showed limited efficacy against established bone tumors, the combination with doxorubicin successfully inhibited bone tumor growth by mounting a localized immune response. This effect was driven by a reduction in ARG1^+^ immunosuppressive myeloid cells and an enhanced sensitivity to T cell targeted therapy within the bone microenvironment.

## CRediT authorship contribution statement

**Xinming Su:** Writing – review & editing, Writing – original draft, Visualization, Validation, Supervision, Software, Resources, Project administration, Methodology, Investigation, Funding acquisition, Formal analysis, Data curation, Conceptualization. **Takayuki Kobayashi:** Writing – review & editing, Validation, Methodology, Formal analysis, Data curation. **Jingyu Xiang:** Writing – review & editing, Methodology, Investigation, Formal analysis, Data curation. **Yalin Xu:** Validation, Resources, Methodology, Investigation, Data curation. **Ayesha N. Shajahan-Haq:** Writing – review & editing, Validation, Methodology, Formal analysis, Data curation. **Mahta Mardani:** Visualization, Validation, Software, Methodology, Data curation. **Suleyman Noordeen:** Methodology, Data curation. **Kaylee O’Donnell:** Methodology, Data curation. **Kristin A. Kwakwa:** Methodology, Data curation. **Dennis Guan:** Validation, Methodology, Data curation. **Gregory C. Fox:** Methodology, Data curation. **Francesca Fontana:** Methodology, Data curation. **Emily Cybulla:** Writing – review & editing, Resources. **Suzanne Bakewell:** Writing – review & editing, Writing – original draft. **Alessandro Vindigni:** Writing – review & editing, Software, Resources, Methodology, Data curation. **Deborah J. Veis:** Writing – review & editing, Resources. **Samuel Achilefu:** Writing – review & editing, Resources. **Gregory M. Lanza:** Writing – review & editing, Resources, Conceptualization. **Katherine N. Weilbaecher:** Writing – review & editing, Writing – original draft, Supervision, Resources, Project administration, Investigation, Funding acquisition, Conceptualization.

## Declaration of competing interest

The authors declare the following financial interests/personal relationships which may be considered as potential competing interests: Katherine N. Weilbaecher reports financial support was provided by Novartis Pharmaceutical. If there are other authors, they declare that they have no known competing financial interests or personal relationships that could have appeared to influence the work reported in this paper.

## References

[1] Kastan M.B., Bartek J. (2004). Cell-cycle checkpoints and cancer. Nature.

[2] Weinberg R.A. (1995). The retinoblastoma protein and cell cycle control. Cell.

[3] Portman N., Alexandrou S., Carson E., Wang S., Lim E., Caldon C.E. (2019). Overcoming CDK4/6 inhibitor resistance in ER-positive breast cancer. Endocr. Relat. Cancer.

[4] Lynce F., Shajahan-Haq A.N., Swain S.M. (2018). CDK4/6 inhibitors in breast cancer therapy: current practice and future opportunities. Pharmacol. Ther..

[5] Fassl A., Sicinski P. (2020). Chemotherapy and CDK4/6 Inhibition in Cancer Treatment: timing is everything. Cancer Cell.

[6] E. Boopathi, R. Birbe, S.A. Shoyele, R.B. Den, C. Thangavel, Bone Health Management in the Continuum of Prostate Cancer Disease, Cancers (Basel) 14(17) (2022). 10.3390/cancers14174305 PMC9455007 36077840

[7] Ihle C.L., Wright-Hobart S.J., Owens P. (2022). Therapeutics targeting the metastatic breast cancer bone microenvironment. Pharmacol. Ther..

[8] Pagnotti G.M., Trivedi T., Mohammad K.S. (2022). Translational strategies to Target Metastatic Bone Disease. Cells.

[9] Joseph G.J., Johnson D.B., Johnson R.W. (2023). Immune checkpoint inhibitors in bone metastasis: Clinical challenges, toxicities, and mechanisms. J Bone Oncol.

[10] Castello A., Lopci E. (2019). Response assessment of bone metastatic disease: seeing the forest for the trees RECIST, PERCIST, iRECIST, and PCWG-2. Q. J. Nucl. Med. Mol. Imaging.

[11] Welte T., Rosen J.M., Zhang X.H. (2016). Fatal attraction: TICs and MDSCs. Cell Cycle.

[12] X. Hao, Y. Shen, N. Chen, W. Zhang, E. Valverde, L. Wu, H.L. Chan, Z. Xu, L. Yu, Y. Gao, I. Bado, L.N. Michie, C.H. Rivas, L.B. Dominguez, S. Aguirre, B.C. Pingel, Y.H. Wu, F. Liu, Y. Ding, D.G. Edwards, J. Liu, A. Alexander, N.T. Ueno, P.R. Hsueh, C.Y. Tu, L.C. Liu, S.H. Chen, M.C. Hung, B. Lim, X.H. Zhang, Osteoprogenitor-GMP crosstalk underpins solid tumor-induced systemic immunosuppression and persists after tumor removal, Cell Stem Cell 30(5) (2023) 648-664 e8. 10.1016/j.stem.2023.04.005 PMC10165729 37146584

[13] Pascual T., Fernandez-Martinez A., Agrawal Y., Pfefferle A.D., Chic N., Braso-Maristany F., Gonzalez-Farre B., Pare L., Villacampa G., Saura C., Hernando C., Munoz M., Galvan P., Gonzalez-Farre X., Oliveira M., Gil-Gil M., Ciruelos E., Villagrasa P., Gavila J., Prat A., Perou C.M. (2024). Cell-cycle inhibition and immune microenvironment in breast cancer treated with ribociclib and letrozole or chemotherapy. npj Breast Cancer.

[14] Deng J., Wang E.S., Jenkins R.W., Li S., Dries R., Yates K., Chhabra S., Huang W., Liu H., Aref A.R., Ivanova E., Paweletz C.P., Bowden M., Zhou C.W., Herter-Sprie G.S., Sorrentino J.A., Bisi J.E., Lizotte P.H., Merlino A.A., Quinn M.M., Bufe L.E., Yang A., Zhang Y., Zhang H., Gao P., Chen T., Cavanaugh M.E., Rode A.J., Haines E., Roberts P.J., Strum J.C., Richards W.G., Lorch J.H., Parangi S., Gunda V., Boland G.M., Bueno R., Palakurthi S., Freeman G.J., Ritz J., Haining W.N., Sharpless N.E., Arthanari H., Shapiro G.I., Barbie D.A., Gray N.S., Wong K.K. (2018). CDK4/6 Inhibition Augments Antitumor Immunity by Enhancing T-cell Activation. Cancer Discov..

[15] Cai X., Yin G., Chen S., Tacke F., Guillot A., Liu H. (2024). CDK4/6 inhibition enhances T-cell immunotherapy on hepatocellular carcinoma cells by rejuvenating immunogenicity. Cancer Cell Int..

[16] Scirocchi F., Scagnoli S., Botticelli A., Di Filippo A., Napoletano C., Zizzari I.G., Strigari L., Tomao S., Cortesi E., Rughetti A., Marchetti P., Nuti M. (2022). Immune effects of CDK4/6 inhibitors in patients with HR(+)/HER2(-) metastatic breast cancer: Relief from immunosuppression is associated with clinical response. EBioMedicine.

[17] Hurvitz S.A., Martin M., Press M.F., Chan D., Fernandez-Abad M., Petru E., Rostorfer R., Guarneri V., Huang C.S., Barriga S., Wijayawardana S., Brahmachary M., Ebert P.J., Hossain A., Liu J., Abel A., Aggarwal A., Jansen V.M., Slamon D.J. (2020). Potent Cell-Cycle Inhibition and Upregulation of Immune Response with Abemaciclib and Anastrozole in neoMONARCH, phase II Neoadjuvant Study in HR(+)/HER2(-) Breast Cancer. Clin. Cancer Res..

[18] Smith M.C., Luker K.E., Garbow J.R., Prior J.L., Jackson E., Piwnica-Worms D., Luker G.D. (2004). CXCR4 regulates growth of both primary and metastatic breast cancer. Cancer Res..

[19] Su X., Esser A.K., Amend S.R., Xiang J., Xu Y., Ross M.H., Fox G.C., Kobayashi T., Steri V., Roomp K., Fontana F., Hurchla M.A., Knolhoff B.L., Meyer M.A., Morgan E.A., Tomasson J.C., Novack J.S., Zou W., Faccio R., Novack D.V., Robinson S.D., Teitelbaum S.L., DeNardo D.G., Schneider J.G., Weilbaecher K.N. (2016). Antagonizing Integrin beta3 increases Immunosuppression in Cancer. Cancer Res..

[20] Su X., Xu Y., Fox G.C., Xiang J., Kwakwa K.A., Davis J.L., Belle J.I., Lee W.C., Wong W.H., Fontana F., Hernandez-Aya L.F., Kobayashi T., Tomasson H.M., Su J., Bakewell S.J., Stewart S.A., Egbulefu C., Karmakar P., Meyer M.A., Veis D.J., DeNardo D.G., Lanza G.M., Achilefu S., Weilbaecher K.N. (2021). Breast cancer-derived GM-CSF regulates arginase 1 in myeloid cells to promote an immunosuppressive microenvironment. J. Clin. Invest..

[21] Su X., Floyd D.H., Hughes A., Xiang J., Schneider J.G., Uluckan O., Heller E., Deng H., Zou W., Craft C.S., Wu K., Hirbe A.C., Grabowska D., Eagleton M.C., Townsley S., Collins L., Piwnica-Worms D., Steinberg T.H., Novack D.V., Conley P.B., Hurchla M.A., Rogers M., Weilbaecher K.N. (2012). The ADP receptor P2RY12 regulates osteoclast function and pathologic bone remodeling. J. Clin. Invest..

[22] Ianevski A., He L., Aittokallio T., Tang J. (2017). SynergyFinder: a web application for analyzing drug combination dose-response matrix data. Bioinformatics.

[23] Weilbaecher K.N., Guise T.A., McCauley L.K. (2011). Cancer to bone: a fatal attraction. Nat. Rev. Cancer.

[24] Miller C.W., Aslo A., Won A., Tan M., Lampkin B., Koeffler H.P. (1996). Alterations of the p53, Rb and MDM2 genes in osteosarcoma. J. Cancer Res. Clin. Oncol..

[25] Wadayama B., Toguchida J., Shimizu T., Ishizaki K., Sasaki M.S., Kotoura Y., Yamamuro T. (1994). Mutation spectrum of the retinoblastoma gene in osteosarcomas. Cancer Res..

[26] Suski J.M., Braun M., Strmiska V., Sicinski P. (2021). Targeting cell-cycle machinery in cancer. Cancer Cell.

[27] Alvarez-Fernandez M., Malumbres M. (2020). Mechanisms of Sensitivity and Resistance to CDK4/6 Inhibition. Cancer Cell.

[28] Vermeulen K., Van Bockstaele D.R., Berneman Z.N. (2003). The cell cycle: a review of regulation, deregulation and therapeutic targets in cancer. Cell Prolif..

[29] Li Z., Zou W., Zhang J., Zhang Y., Xu Q., Li S., Chen C. (2020). Mechanisms of CDK4/6 Inhibitor Resistance in Luminal Breast Cancer. Front. Pharmacol..

[30] Guido C., Whitaker-Menezes D., Capparelli C., Balliet R., Lin Z., Pestell R.G., Howell A., Aquila S., Ando S., Martinez-Outschoorn U., Sotgia F., Lisanti M.P. (2012). Metabolic reprogramming of cancer-associated fibroblasts by TGF-beta drives tumor growth: connecting TGF-beta signaling with “Warburg-like” cancer metabolism and L-lactate production. Cell Cycle.

[31] Zhang H., Liu L., Liu J., Dang P., Hu S., Yuan W., Sun Z., Liu Y., Wang C. (2023). Roles of tumor-associated macrophages in anti-PD-1/PD-L1 immunotherapy for solid cancers. Mol. Cancer.

[32] Baas M., Besancon A., Goncalves T., Valette F., Yagita H., Sawitzki B., Volk H.D., Waeckel-Enee E., Rocha B., Chatenoud L., You S. (2016). TGFbeta-dependent expression of PD-1 and PD-L1 controls CD8(+) T cell anergy in transplant tolerance. Elife.

[33] J.C. Jennifer Gao, Mallorie Fiero, Shenghui Tang, Suparna B. Wedam, Melanie E. Royce, Tatiana Michelle Prowell, Elaine Chang, Mirat Shah, Preeti Narayan, Christy Osgood, Nicole Gormley, Tamy Kim, Richard Pazdur, Paul Gustav Kluetz, and Laleh Amiri-Kordestani, Overall survival in patients with HR+/HER2- advanced or metastatic breast cancer treated with a cyclin-dependent kinase 4/6 inhibitor plus an aromatase inhibitor: A US Food and Drug Administration pooled analysis, Journal of Clinical Oncology 43 (2025).

[34] Voorwerk L., Slagter M., Horlings H.M., Sikorska K., van de Vijver K.K., de Maaker M., Nederlof I., Kluin R.J.C., Warren S., Ong S., Wiersma T.G., Russell N.S., Lalezari F., Schouten P.C., Bakker N.A.M., Ketelaars S.L.C., Peters D., Lange C.A.H., van Werkhoven E., van Tinteren H., Mandjes I.A.M., Kemper I., Onderwater S., Chalabi M., Wilgenhof S., Haanen J., Salgado R., de Visser K.E., Sonke G.S., Wessels L.F.A., Linn S.C., Schumacher T.N., Blank C.U., Kok M. (2019). Immune induction strategies in metastatic triple-negative breast cancer to enhance the sensitivity to PD-1 blockade: the TONIC trial. Nat. Med..

[35] Wang J., Hu C., Wang J., Shen Y., Bao Q., He F., Wang H., Gong L., Liu Z., Hu F., Liang J., Zhou Q., Wei L., Wen J., Zhang W. (2019). Checkpoint Blockade in Combination with Doxorubicin Augments Tumor Cell Apoptosis in Osteosarcoma. J. Immunother..

[36] Yang M., Liu P., Wang K., Glorieux C., Hu Y., Wen S., Jiang W., Huang P. (2017). Chemotherapy induces tumor immune evasion by upregulation of programmed cell death ligand 1 expression in bone marrow stromal cells. Mol. Oncol..

[37] Lee J.S., Hackbart H., Cui X., Yuan Y. (2023). CDK4/6 Inhibitor Resistance in Hormone Receptor-positive Metastatic Breast Cancer: Translational Research, Clinical Trials, and Future Directions. Int. J. Mol. Sci..

[38] Hussein S.A., Saadawy A.H., Badr E., Abdollah M.R.A., Wael N., Allam R.M., Al-Abd A.M., Roncero Sanchez A.M., Tolba M.F. (2025). The landscape of cyclin-dependent kinase 4/6 inhibitors in solid malignancies: emphasis on immunotherapy combinatorial strategies. Med. Oncol..

[39] Llinas-Bertran A., Butjosa-Espin M., Barberi V., Seoane J.A. (2025). Multimodal data integration in early-stage breast cancer. Breast.

[40] Mattarollo S.R., Loi S., Duret H., Ma Y., Zitvogel L., Smyth M.J. (2011). Pivotal role of innate and adaptive immunity in anthracycline chemotherapy of established tumors. Cancer Res..

[41] Francis A.M., Alexander A., Liu Y., Vijayaraghavan S., Low K.H., Yang D., Bui T., Somaiah N., Ravi V., Keyomarsi K., Hunt K.K. (2017). CDK4/6 Inhibitors Sensitize Rb-positive Sarcoma Cells to Wee1 Kinase Inhibition through Reversible Cell-Cycle arrest. Mol. Cancer Ther..

[42] Goel S., DeCristo M.J., Watt A.C., BrinJones H., Sceneay J., Li B.B., Khan N., Ubellacker J.M., Xie S., Metzger-Filho O., Hoog J., Ellis M.J., Ma C.X., Ramm S., Krop I.E., Winer E.P., Roberts T.M., Kim H.J., McAllister S.S., Zhao J.J. (2017). CDK4/6 inhibition triggers anti-tumour immunity. Nature.

[43] Hortobagyi G.N., Stemmer S.M., Burris H.A., Yap Y.S., Sonke G.S., Hart L., Campone M., Petrakova K., Winer E.P., Janni W., Conte P., Cameron D.A., Andre F., Arteaga C.L., Zarate J.P., Chakravartty A., Taran T., Le Gac F., Serra P., O'Shaughnessy J. (2022). Overall Survival with Ribociclib plus Letrozole in Advanced Breast Cancer. N. Engl. J. Med..

[44] Hart L.L., Im S.A., Tolaney S.M., Campone M., Pluard T., Sousa B., Freyer G., Decker T., Kalinsky K., Sopher G., Gao M., Hu H., Kuemmel S. (2025). Efficacy, safety, and patient-reported outcomes across young to older age groups of patients with HR+/HER2- advanced breast cancer treated with ribociclib plus endocrine therapy in the randomized MONALEESA-2, -3, and -7 trials. Eur. J. Cancer.

