# Supplementary material for: CDK4/6 inhibitor ribociclib and doxorubicin combination treatment inhibits breast cancer bone metastasis and enhances T-cell targeted therapy
Source: J Bone Oncol. 2026 Apr 25;58:100765. doi: 10.1016/j.jbo.2026.100765 (PMC13139965; doi:10.1016/j.jbo.2026.100765)
Supplement: Supplementary Data 1 [file mmc1.docx]

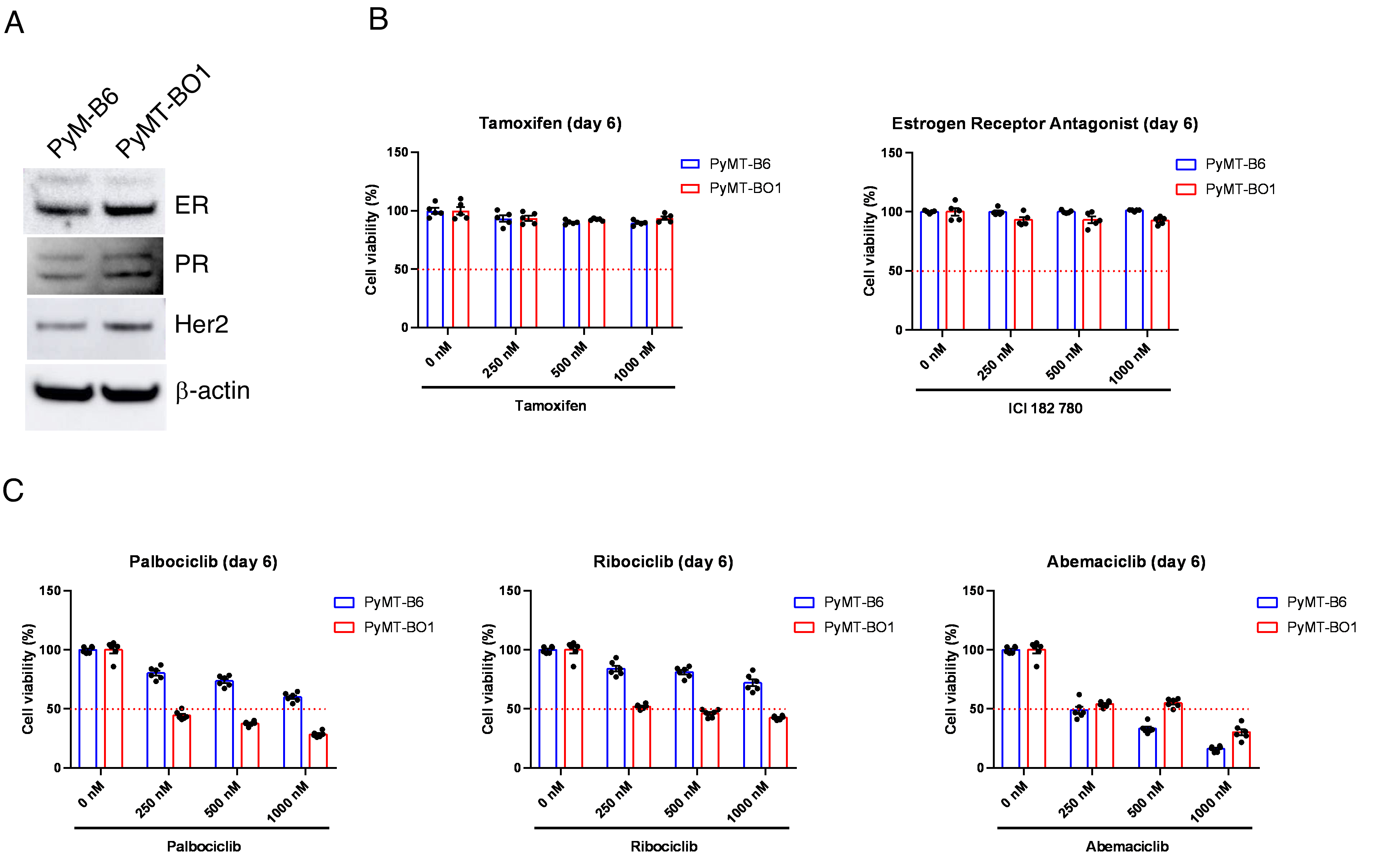


**Supplemental Figure 1. PyMT-BO1 cells express ER, PR, and HER2 receptors but exhibit resistance to tamoxifen treatment in vitro.**

**(A)** Western blot analysis of cell lysates from PyMT-B6 and PyMT-BO1 cells to assess expression levels of estrogen receptor (ER), progesterone receptor (PR), and HER2. **(B, C)** Proliferative response of PyMT-B6 and PyMT-BO1 cells following treatment with **(B)** estrogen receptor-targeted agents (tamoxifen and Fulvestrant) or **(C)** CDK4/6 inhibitors. Cell proliferation was evaluated on day 6 using a crystal violet assay.


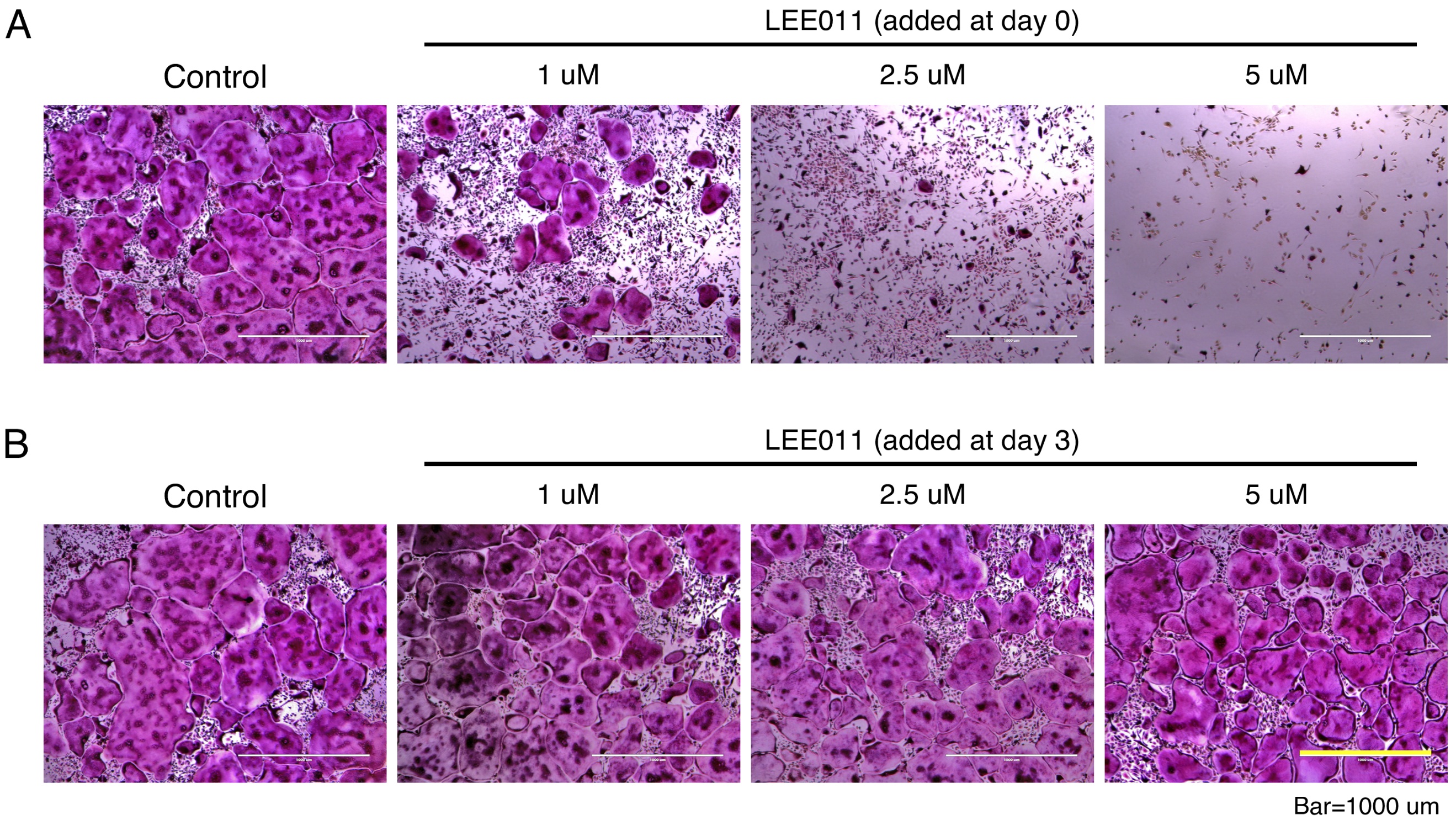


**Supplemental Figure 2. Single agent LEE011 treatment inhibits osteoclast development but has minimal effect on pre-osteoclast fusion.**

Bone marrow macrophages (BMMs) from eight-week-old female C57BL/6J mice were cultured with M-CSF (10 ng/mL) and RANKL (50 ng/mL) for five days to induce osteoclastogenesis. Ribociclib (LEE011) was added at either day 0 **(A)** to assess early differentiation or day 3 **(B)** to evaluate late-stage pre-osteoclast fusion. Osteoclast differentiation was assessed by TRAP staining on day 5. Representative images were captured at 4x magnification; scale bar = 1000 μm.


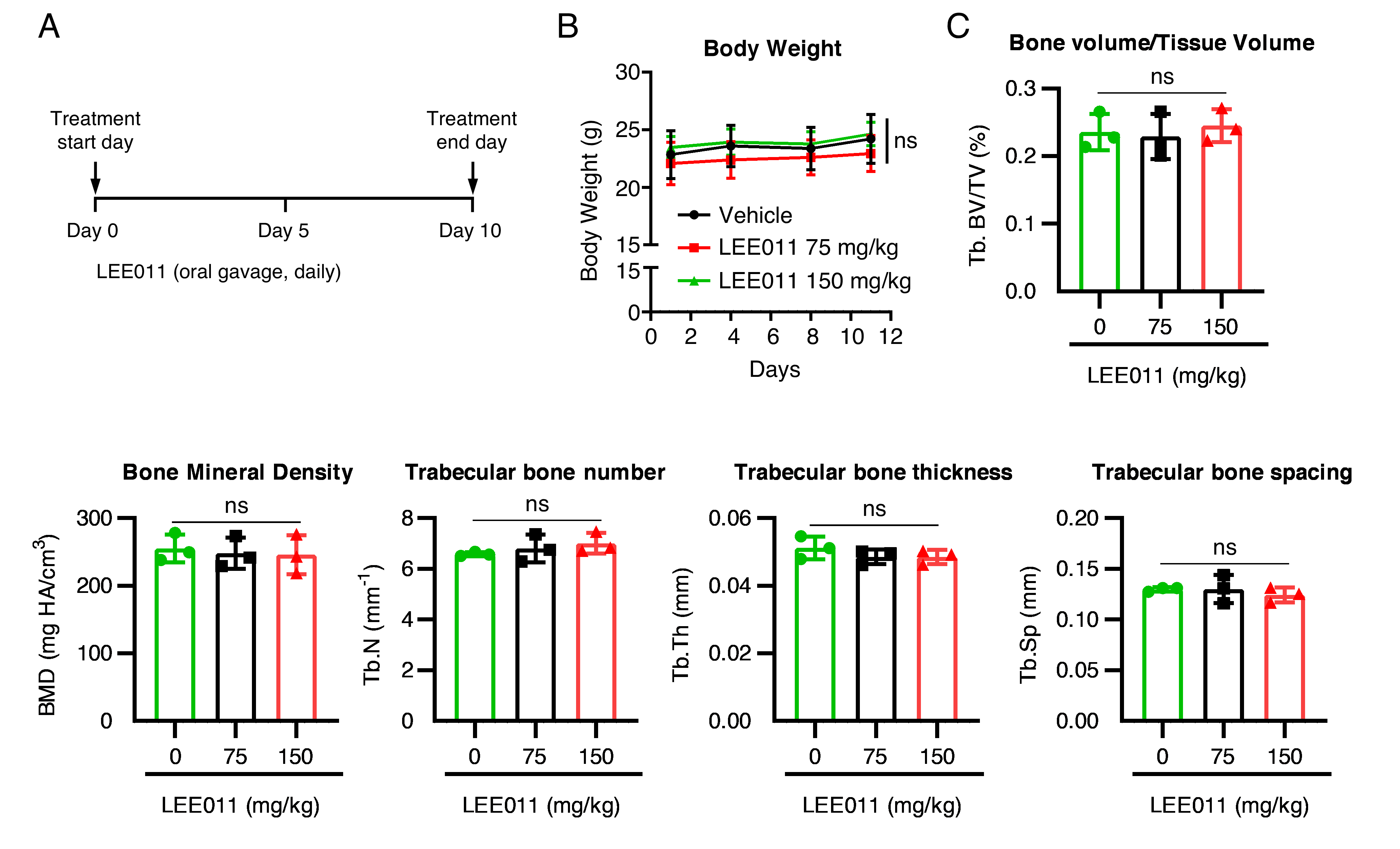


**Supplemental Figure 3. Single agent LEE011 treatment does not alter bone density parameters.**

**(A)** Experimental timeline for eight-week-old female C57BL/6J mice treated with vehicle or ribociclib (LEE011) (75 mg/kg or 150 mg/kg) via oral gavage for 10 days. **(B)** Body weight change recorded over the treatment period. **(C)** On day 11, mice were euthanized for microCT analysis of the distal femur. Representative parameters include trabecular bone volume fraction (BV/TV), bone mineral density (BMD), trabecular number (Tb.N), thickness (Tb.Th), and spacing (Tb.Sp). Data are presented as mean ± SEM. ns = no significant differences, by one-way ANOVA with Tukey’s post-hoc test.


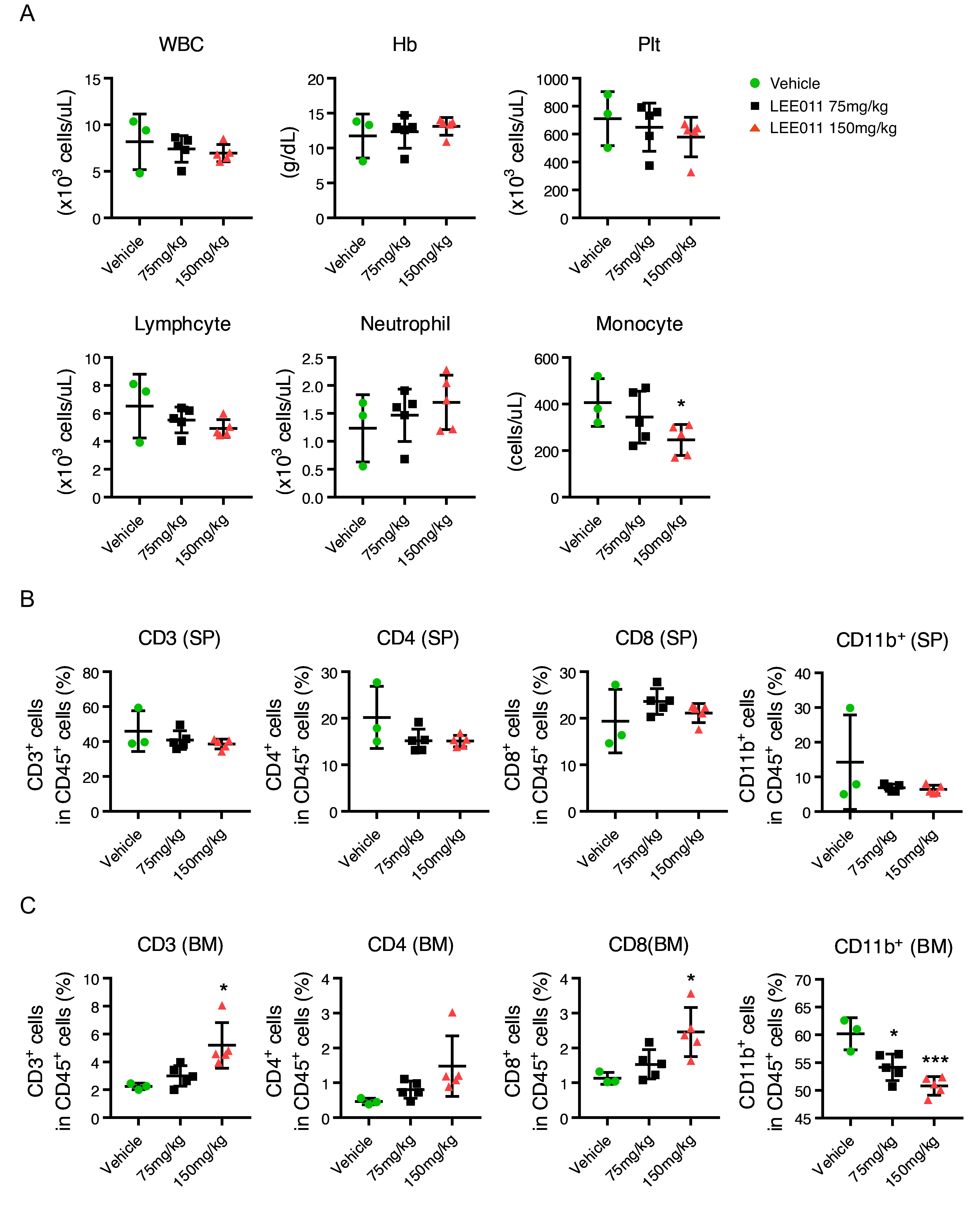


**Supplemental Figure 4. Dose-dependent effects of LEE011 on systemic immune populations in non-tumor bearing mice.**

Eight-week-old female C57BL/6J mice were treated with vehicle or ribociclib (LEE011) at 75 mg/kg or 150 mg/kg via oral gavage for 10 days. On day 11, mice were euthanized for complete blood count **(A)** and flow cytometric analysis (FACS) of the spleen **(B)** and bone marrow **(C)**. Data are presented as the mean ± SEM. **P* < 0.05, ***P* < 0.01, ****P* < 0.001, by two-tailed, unpaired Student’s t test with Welch’s correction.

**
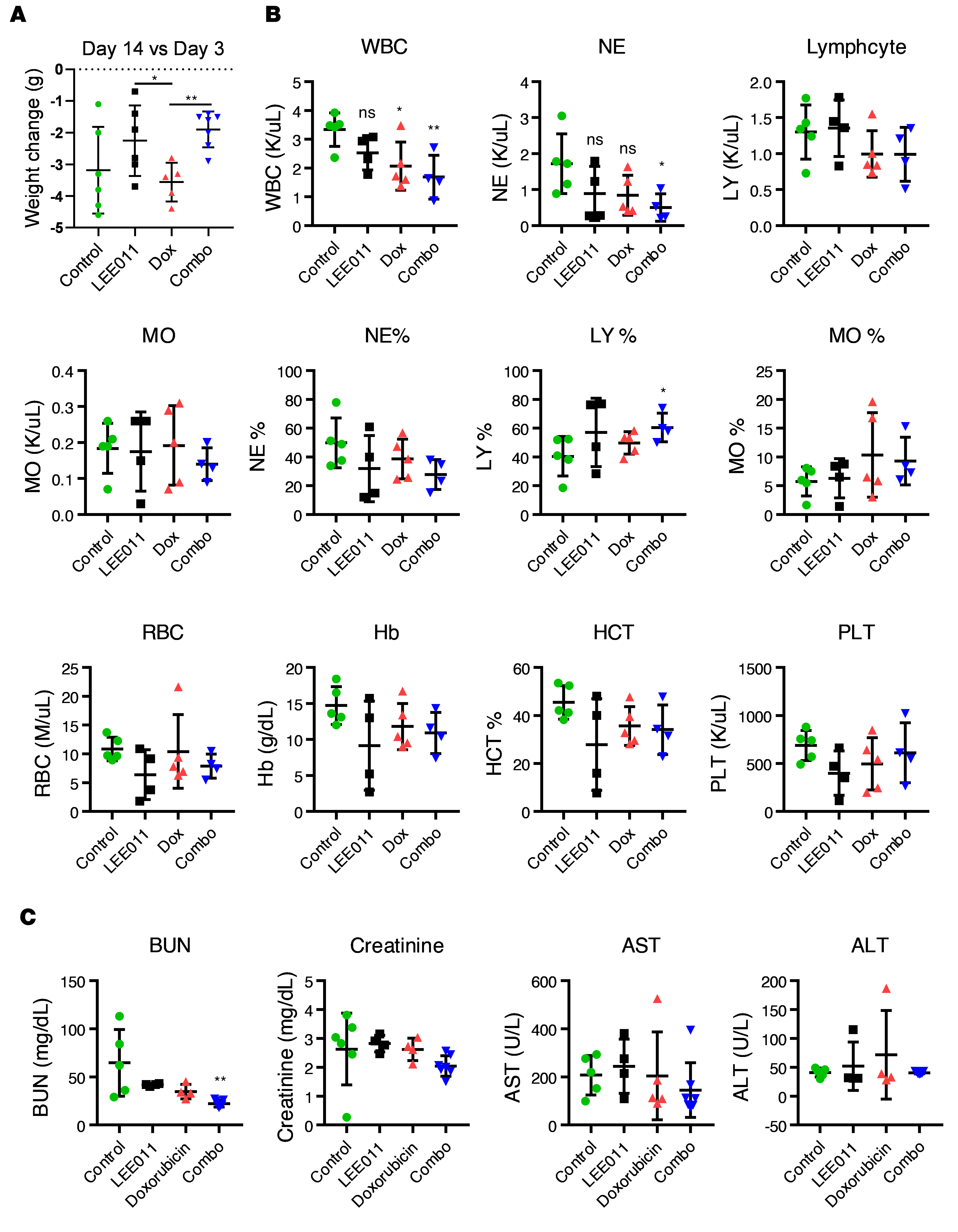
**

**Supplemental Figure 5. Effect of LEE011 and doxorubicin combined treatment on blood count and organ function.**

PyMT-BO1-GFP-Luc breast tumor cells (1 × 10^5^) were intracardially injected into 6-week-old female C57BL/6J mice. LEE011 treatment was administered by oral gavage from day 4 to day 13. Doxorubicin (4mg/kg) was administered via i.v. injection on day 5 and day 10. On day 14, mice were dissected to harvest blood and organ tissue. **(A)** Total body weight lost (g) from day 3 to day 14. **(B)** Blood count analysis, **(C)** Assessment of kidney (BUN, Creatinine) and liver (AST, ALT) function. Data are shown as the mean ± SEM. **P* < 0.05, ***P* < 0.01, by two-tailed, unpaired Student’s t test with Welch’s correction.


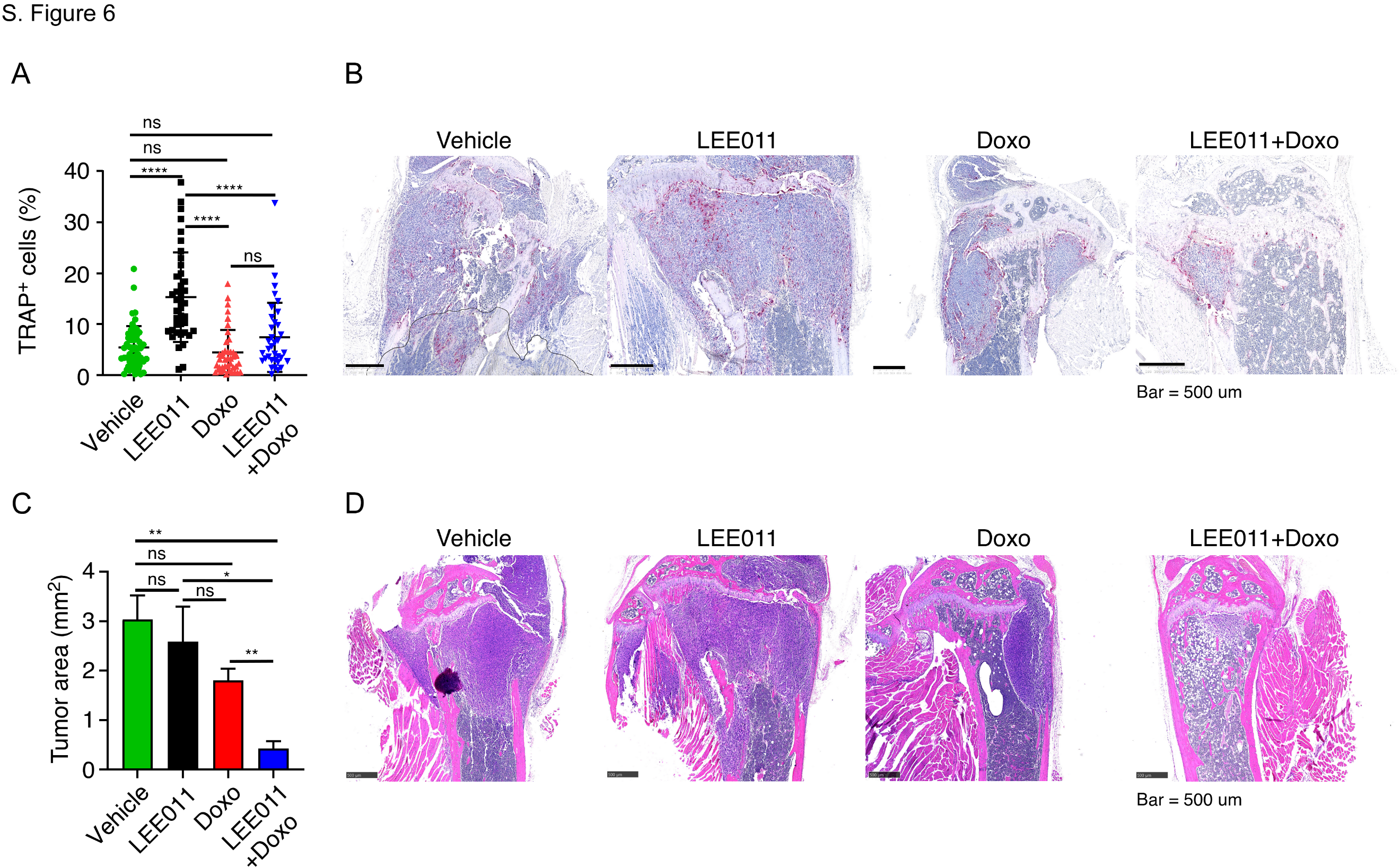


**Supplemental Figure 6. Combined LEE011 and doxorubicin treatment reverses LEE011-induced osteoclast activity and reduces bone tumor burden.**

PyMT-BO1-GFP-Luc breast tumor cells (1 × 10^5^) were intracardially injected into 6-week-old female C57BL/6J mice. LEE011 treatment was administrated by oral gavage from day 4 to day 13. Doxorubicin (4mg/kg) was administered via i.v. injection on day 5 and day 10. On day 14, femur and tibia were harvested for histological analysis. **(A, B)** Representative TRAP staining and quantification of TRAP+ osteoclasts, expressed as the percentage of TRAP+ cells per region of interest (ROI). **(C, D)** Representative H&E staining and quantification of tumor area per bone. All images were captured at 5x magnification; scale bar = 500 μm. Data are shown as the mean ± SEM. *P < 0.05, **P < 0.01, ***P < 0.001, ****P < 0.0001, by 2-tailed, unpaired Student’s t test with Welch’s correction.


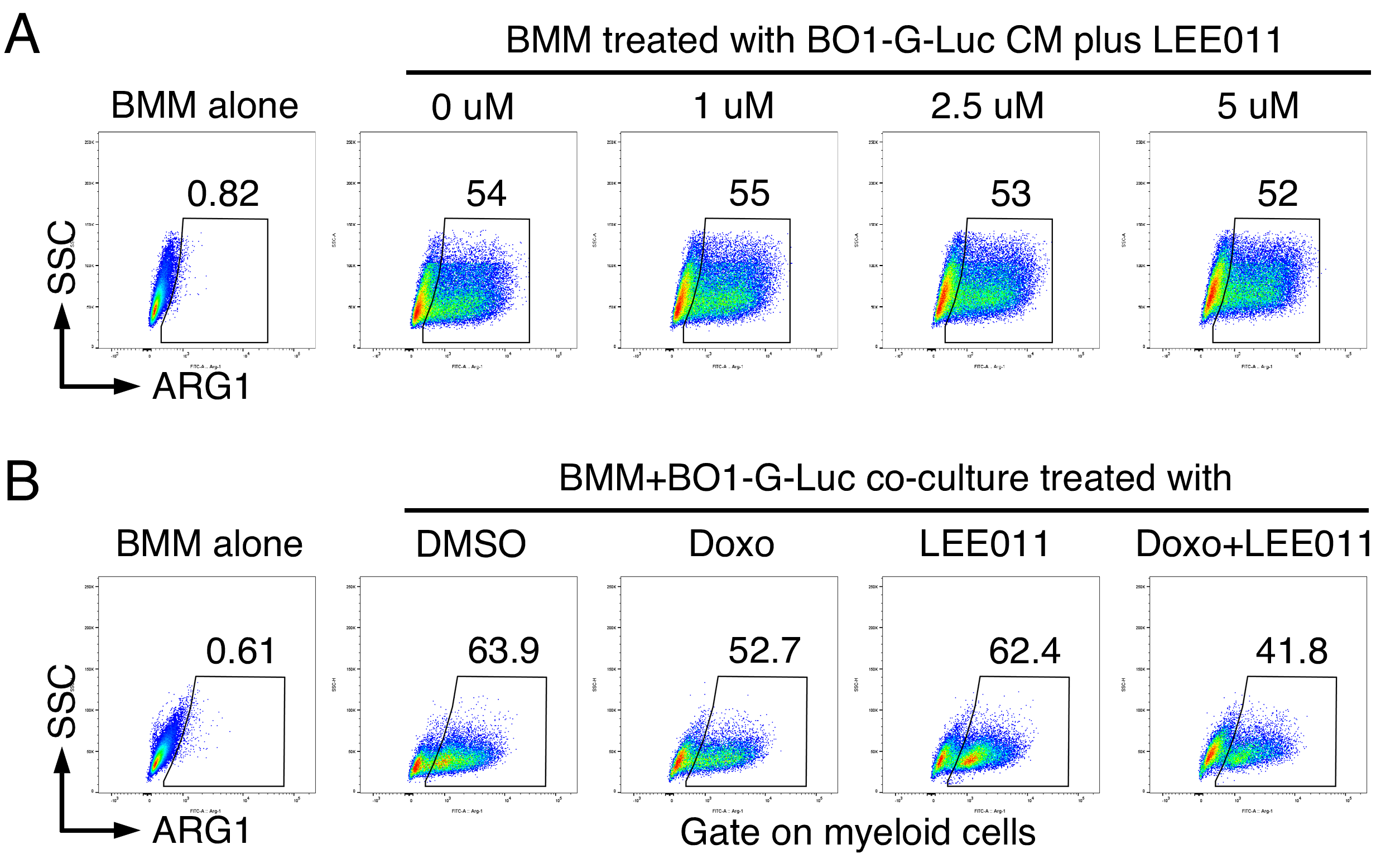


**Supplemental Figure 7. LEE011 and doxorubicin combined treatment decreases tumor cell induced ARG1 expression in bone marrow macrophages.**

**(A)** Bone marrow macrophages from 8 weeks-old C57BL/6J female mice were treated with PyMT-BO1 cell conditioned media plus LEE011 for 24 hour, ARG1 expression analyzed by FACS. **(B)** BMMs co-cultured with PyMT-BO1 tumor cells at 1:1 ratio in 6-well plate, the co-culture system was treated with LEE011, doxorubicin, or LEE011 plus doxorubicin for 24 hour, ARG1 expression was analyzed by FACS.

**Supplemental Table 1. Mouse Flow Cytometry and IHC Antibodies**

| **Antigen** | **Clone** | **Source** | **Fluorophore** | **Dilution** |
| --- | --- | --- | --- | --- |
| CD45 | 30-F11 | eBioscience | PE-Cy7, APC-eFluor780 | 1:400 |
| CD11b | M1/70 | eBioscience | APC, PE, Alexa Fluor 700 | 1:400 |
| Ly6G | 1A8 | BioLegend | PE, APC | 1:400 |
| Ly6C | HK1.4 | eBioscience | PerCP-Cy5.5 | 1:400 |
| Gr-1 | RB6-BC5 | BioLegend | FITC, PerCP-Cy5.5 | 1:400 |
| F4/80 | BM8 | eBioscience | PE-Cy5 | 1:400 |
| CD206 | 19.2 | eBioscience | FITC, PerCP-Cy5.5 | 1:50 |
| MHCII | M5/114.15.2 | eBioscience, BD | eFluor450, PE | 1:400 |
| CD3e | 145-2C11 | eBioscience, BD Horizon | PerCP-Cy5.5, APC, BV711 | 1:200 |
| CD4 | RM4-4 | eBioscience | FITC | 1:200 |
| CD8a | 53-6.7 | BD Horizon | BUV395, PE | 1:200 |
| ARG1 | D4E3M | Cell Signaling | Unconjugated | 1:200 |
| Ki67 | D3B5 | Cell Signaling | Unconjugated | 1:200 |
